# A Rapid Cortical Learning Process Supporting Students’ Knowledge Construction During Real Classroom Teaching

**DOI:** 10.1002/advs.202416610

**Published:** 2025-02-07

**Authors:** Xiaodan Feng, Xinran Xu, Zhaonan Meng, Jiahao Jiang, Miao Pei, Yonghe Zheng, Chunming Lu

**Affiliations:** ^1^ State Key Laboratory of Cognitive Neuroscience and Learning and IDG/McGovern Institute for Brain Research Beijing Normal University Beijing 100875 China; ^2^ Center for Teacher Education Research Beijing Normal University Beijing 100875 China; ^3^ Research Institute of Science Education Beijing Normal University Beijing 100875 China

**Keywords:** blended teaching, classroom teaching, functional near‐infrared spectroscopy, knowledge construction, neural synchronization, rapid cortical learning

## Abstract

Classroom teaching is essential for cognitive development and cultural evolution, yet its neurocognitive mechanisms remain unclear. Here, this is explored in a university graduate course by combining wearable functional near‐infrared spectroscopy (fNIRS) and machine learning models. The results show that blended teaching involving both students’ recalling and teachers’ lecturing leads to better learning outcomes than lecturing alone. Moreover, during the same lecturing phase, blended teaching induces knowledge construction in the middle frontal cortex (MFC), while lecturing alone induces knowledge representation in the right temporoparietal junction (TPJ), with the former significantly correlating with the final learning outcomes. Additionally, the MFC's construction begins during earlier recalling but is significantly facilitated by later lecturing. Finally, when teacher's TPJ activity precedes that of students’ MFC, significant teacher–student neural synchronization is observed during lecturing of blended teaching and is correlated with learning outcomes. These findings suggest that, in the real classroom teaching, the MFC serves as a hub of a rapid cortical learning process, supporting knowledge construction through a projection from the teacher's TPJ.

## Graphical abstract

There is growing interest in understanding how classroom teaching works, but previous theories and studies leave a gap in the neurocognitive mechanisms involved in real classroom teaching. Here we show that during real classroom teaching, students' middle frontal cortex serves as a hub of a rapid cortical learning process, supporting their knowledge construction through a projection from teacher's temporoparietal junction.

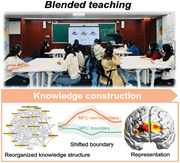

## 1 Introduction

John Amos Comenius once stated: “It is nevertheless better that the young should be taught together and in large classes.”^[^ ^1^ ^]^ This is because classroom teaching plays a crucial role in human cognitive development and societal cultural evolution.^[^ ^2^, ^3^ ^]^ Over the past centuries, there has been persistent interest and a growing demand to understand how classroom teaching functions, with the goal of improving the practices of teaching and learning.^[^ ^4^, ^5^ ^]^ Unfortunately, many earlier pedagogical theories were based more on reflection or speculation, while extensive empirical studies were primarily confined to human individual or animal learning in laboratory settings.^[^ ^6^, ^7^ ^]^ As a result, a notable gap remains in our understanding of the neurocognitive mechanisms that underpin naturalistic teaching in real classrooms.

The internal neurocognitive processes of students’ learning in the classroom can provide insights into the underlying mechanisms of effective teaching. According to Wittrock's generative learning theory, learning is an active and dynamic process in which students construct meaning, rather than passively receiving and recording incoming sensory information.^[^ ^8^, ^9^ ^]^ More specifically, during knowledge construction, individuals selectively focus on knowledge events in learning situations and link these new knowledge events with existing knowledge, concepts, and background information. This is achieved through dynamically constructing and reorganizing the boundaries and internal relational networks of knowledge events.^[^ ^9^, ^10^ ^]^ According to this theory, there are two crucial aspects in knowledge construction, that is, the relationship between knowledge events should be reorganized, and/or the boundaries of knowledge events should be shifted. However, there has been a long‐standing debate on whether classroom teaching facilitates students’ construction of knowledge. Some perspectives suggest that, during classroom teaching, knowledge construction may not be a primary aim for students and teachers, as they may lack sufficient time to construct knowledge during the learning–teaching process. Therefore, teachers mostly transmit ready‐made knowledge in a priori designed sequence or structure.^[^ ^11^ ^]^ Conversely, other perspectives imply that knowledge is often well‐structured in the educational system to facilitate students’ construction, enabling them to quickly apply knowledge in other contexts.^[^ ^12^ ^]^ This debate is further complicated by conflicting pedagogical theories, with some advocating for direct instruction, such as the traditional lecturing, where knowledge is pre‐structured,^[^ ^13^ ^]^ while others argue that blending high‐structured teacher‐led lecturing with low‐structured student‐led inquiry is the most effective approach to enhancing the quality of classroom teaching.^[^ ^14^, ^15^, ^16^ ^]^ Despite the widespread use of both teaching methods, the neurocognitive mechanisms that underlie their effectiveness remain poorly understood.

Extensive studies have investigated knowledge construction during human individual learning. For example, previous neuroscientific evidence indicates that naturalistic stimuli typically consist of events with intrinsic structure.^[^ ^17^, ^18^ ^]^ When the neural trace produced by an event during its initial encoding is reinstated during later retrieval,^[^ ^19^, ^20^, ^21^, ^22^ ^]^ the intrinsic structure of knowledge is constructed.^[^ ^23^, ^24^ ^]^ In this sense, knowledge construction entails the re‐coding of prior knowledge events and the reorganization of their relations based on existing schemas,^[^ ^25^ ^]^ ultimately changing both the local conceptual boundaries and the global structure of the knowledge network. Moreover, according to the global–local processing theory, knowledge construction is influenced by how students process global or local attributes of knowledge structures.^[^ ^26^ ^]^ Constructing global attributes involves organizing and integrating new information by understanding the overall knowledge network, while constructing local attributes requires mastering the distinctiveness of specific knowledge events and reassigning their boundaries. The prioritization of these attributes can vary, influencing the manner in which knowledge is constructed.^[^ ^27^ ^]^ Integrating pedagogical speculations on teaching and neuroscientific evidence on knowledge construction, we hypothesize that effective classroom teaching facilitates constructing boundaries and reorganizing relations between events, rather than merely representing the semantics of individual knowledge events in students’ brains. However, it remains unclear whether, how and which attributes of knowledge are constructed in students’ brains during real classroom teaching, and how this relates to their learning outcomes.

Previous neuroscientific studies on both human individual and animal learning suggest that long‐term knowledge representation and construction are likely linked to significant contributions from the hippocampus.^[^ ^28^, ^29^ ^]^ However, recent studies on animals and human individual learners in strictly controlled laboratory settings have identified the rapid formation of cortical engrams during learning, which can support hippocampal‐independent memories within a short period.^[^ ^30^, ^31^, ^32^ ^]^ Additionally, brain regions such as the medial prefrontal cortex, posterior parietal cortex (including temporoparietal junction, TPJ), angular gyrus, and anterior temporal lobe have been implicated in supporting such a rapid cortical learning process through integrating congruent events during wakefulness, independently of hippocampal activity.^[^ ^31^, ^33^, ^34^, ^35^, ^36^ ^]^ Furthermore, patients with frontal lobe lesions struggle to establish relations among knowledge events, thus failing to construct knowledge during retrieval.^[^ ^37^ ^]^ The potential rapid cortical learning process is particularly relevant to classroom teaching because classroom teaching in the educational systems also occurs within a limited time period to avoid fatigue among students. Importantly, the potential role of the rapid cortical learning process can be investigated in real classroom settings using promising noninvasive and wearable neuroimaging techniques such as functional near‐infrared spectroscopy (fNIRS) and electroencephalography (EEG).

Starting around 2012, the successful combination of wearable neuroimaging techniques and the naturalistic free interaction paradigm made it possible to directly study the neurocognitive mechanisms underlying classroom teaching,^[^ ^38^ ^]^ and such studies have gradually emerged. Among these, Holpler et al. conducted the first study between a teacher and a student, showing a close association between teacher–student neural synchronization and learning outcomes.^[^ ^39^ ^]^ This association and the paradigm were well replicated in subsequent studies and even extended to real classroom settings involving one teacher and twelve students.^[^ ^40^, ^41^, ^42^, ^43^, ^44^ ^]^ Moreover, Zheng et al. decomposed the dynamic sequence of teaching processes, demonstrating that several interaction processes such as effective communication, the emergence of the zone of proximal development through interpersonal prediction, and knowledge transmission unfold over time between a teacher and a student, with each supported by a distinct temporal and spatial pattern of teacher–student neural synchronization.^[^ ^42^ ^]^ Further evidence from a study on observational learning indicates that the neural synchronization with a time lag between a model's and an observer's brain activities (i.e., the brain activity of one individual preceding that of the other by seconds) can effectively track the interpersonal prediction process in the observer, whereas the single‐brain activity in the observer alone cannot.^[^ ^45^ ^]^ Although these studies have provided important insights into the neurocognitive processes of teaching and learning, most have focused on external teaching and learning behaviors rather than the internal neurocognitive processes like knowledge representation and construction, which are central to the science of learning over the past centuries. Therefore, to the best of our knowledge, no studies have yet tested the potential role of the rapid cortical learning process in supporting students’ knowledge representation and construction during real classroom teaching.

This study aimed to fill the gap in the literature by investigating the neurocognitive mechanisms of real classroom teaching, combining fNIRS hyperscanning and machine learning techniques. Here, fNIRS was chosen for its clear advantages over other techniques such as funcational magnetic resonance imaging (fMRI) and EEG, particularly in terms of wearability, high tolerance of movement artifacts, and its ability to measure local hemodynamic responses.^[^ ^46^, ^47^ ^]^ During the experiment, we investigated the neurocognitive processes involved in blended teaching, which combined low‐ and high‐structured teaching styles (low‐to‐high, LH), and traditional lecture‐based teaching, which relied solely on the high‐structured teaching style (H‐only). Here, high‐structured teaching corresponds to direct instruction by the teacher (i.e., lecturing), while low‐structured teaching corresponds to students’ recalling of knowledge in a learning group within the classroom. This recalling phase in the LH condition served to activate students’ prior knowledge, providing a cognitive scaffold that might enhance subsequent learning. According to the Donders’ subtraction method,^[^ ^48^ ^]^ the comparison between LH and H‐only conditions allowed us to reveal the specific contribution of the low‐structured teaching style—including the activation of prior knowledge—to learning outcomes in the LH condition compared to that of the H‐only condition. We simultaneously measured brain activity in the bilateral prefrontal cortex (PFC) and right TPJ in the rapid cortical learning system from both teachers and students, along with students’ learning outcomes. According to the theory of knowledge construction, we segmented the knowledge taught by teachers as well as the knowledge recalled by students into knowledge events and further generated the vectorized semantic representations of these knowledge events using a machine learning model, based on which the relations between events (i.e., knowledge structure) were obtained. We examined how LH and H‐only differed in facilitating students’ representation and construction of knowledge, and whether there was a cortical learning system supporting the representation and construction process within the limited teaching period and affecting learning outcomes. We predicted that both the PFC and TPJ would be involved in knowledge representation and construction in the LH condition, but no such a process would occur in the H‐only condition. The brain processing pattern would be consistent with either the global or local hypotheses of knowledge construction, or both. Finally, the time‐lagged teacher–student neural synchronization should play an important role in students’ knowledge construction and correlate with their learning outcomes.

## 2 Results

### 2.1 Experimental Setup

**Figure 1:**
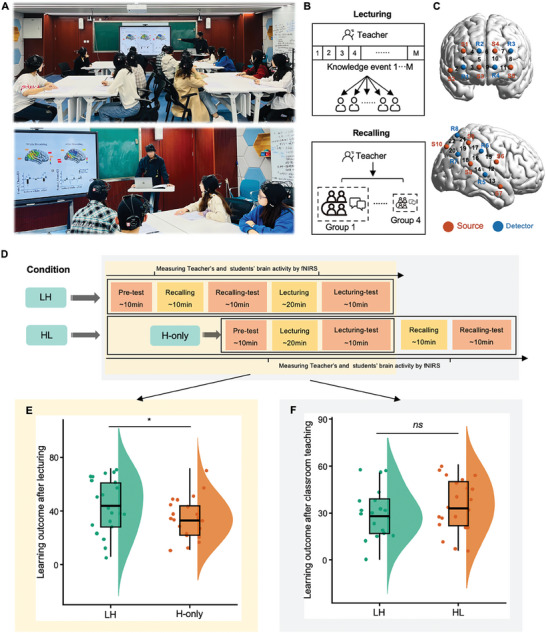
The experimental design and behavioral results of learning outcomes. A) The experimental setup during classroom teaching. B) Experimental paradigm. (Top) Lecturing corresponded to direct instruction by the teacher, who taught the knowledge to students. Students were requested to listen carefully, and no questioning or answering was allowed. (Bottom) Recalling was a form of low‐structured teaching, in which students were asked to freely recall the knowledge within a small learning group. C) fNIRS data acquisition. The optode sets covered the prefrontal cortex and right temporoparietal junction. Measurement channels are marked by numbers. The Montreal Neurological Institute (MNI) coordinates of each channel are provided in Table S1, Supporting Information. S, source; D, detector. D) Experimental design. In the LH condition, students were provided with the written learning materials in a week prior to classroom teaching. During subsequent classroom teaching, students first recalled the knowledge in a small learning group and then listened to the lecture taught by the teacher. In the H‐only condition, classroom teaching occurred immediately upon students' arrival without prior individual learning. In the HL condition, students first experienced the lecturing and followed by recalling phases. Tests were administered before and after each lecturing or recalling phase per condition. Participants underwent both LH and HL with a 1‐week interval between conditions. E) Learning outcomes. Blended teaching showed significantly better learning outcomes than did lecturing alone. F) There was no order effect of blended teaching (LH vs. HL) on behavioral learning outcomes.

A class of a university's graduate course on neuroimaging techniques was selected for this study (**Figure** **1**A). In total, 24 healthy adult students who registered for this course for credits volunteered to participate in this study. Participants also received monetary compensation for their time. According to their self‐report on available time, they were randomly assigned to two lessons, with one lesson involving 16 students, while the other involving eight students (see Experimental Section, Figure 1B,C). In later analyses, the data from the two lessons were combined to increase statistical power (see below) and meanwhile to exclude the potential confounding from teaching contents. The same students in each lesson were requested to participate in both the LH and H‐only conditions, with a 1‐week interval inserted between conditions (Figure 1D). Four teachers with comparable teaching experience (mean = 1.061 years, range = 0.825–1.250 years) were recruited and randomly assigned to the four lessons of the two conditions. To minimize potential carry‐over effects between the LH and H‐only conditions, different learning materials were used for the two conditions, and each teacher was assigned to only one set of materials/conditions. The order of the two conditions were counterbalanced between lessons. These designs ensured that the differences observed between the two conditions were attributable solely to the additional involvement of recalling in the LH condition. Both video and fNIRS recordings were obtained during the entire experiment within the classroom. Three students were excluded due to incomplete data collection in the LH condition, leaving 21 students with valid data.

In the LH condition, students were provided with learning materials in written form a week in advance and notified that they needed to take the lesson 1 week later. During the lesson, these students arrived in the classroom and were organized into small groups, each consisting of two to four members. Each student was requested to recall their knowledge they had learned from the learning materials and report the recalled knowledge aloud to the other members of the same group (i.e., the recalling phase, Figure 1B). This process took about 10 min, during which they were not allowed to take notes or communicate with each other freely (Figure 1D, see Experimental Section). Next, the teacher taught the knowledge contained in the learning materials to these students in a lecturing mode within 20 min (i.e., the lecturing phase, Figure 1B). During this process, students were instructed to listen carefully, and no questioning or answering was allowed. They were permitted to take notes but prohibited from interacting with other students. Students completed behavioral tests on the knowledge taught immediately before individual learning (baseline‐test), before and after recalling (pre‐test and recalling‐test), and after lecturing (lecturing‐test). These tests were self‐paced, with 18 multiple‐choice questions for each test spanning a variety of levels of learning outcomes such as remembering, understanding and applying (six questions per level) according to Bloom's Taxonomy.^[^ ^49^ ^]^

In the H‐only condition, the lecturing took place immediately when the students arrived in the class without any prior exposure to learning materials or the recalling process (Figure 1D). The teacher taught the knowledge in the same way as in the lecturing phase of the LH condition and the students were requested to listen carefully. No questioning or answering was allowed. Students were tested before and after teacher's lecturing (pre‐test and lecturing‐test).

Finally, to test the order effect of blended teaching, that is, whether performing low‐structured teaching before and after the high‐structured teaching would lead to differences in the teaching processes and learning outcomes, students were requested to freely recall the knowledge in the same way as in the LH condition immediately after teacher's lecturing, generating an additional HL condition (Figure 1D). Students were tested after their recalling (recalling‐test).

### 2.2 Blended Teaching Led to Better Learning Outcomes than Lecturing Alone

To confirm whether blended teaching (i.e., the LH condition) led to better learning outcomes than did lecturing alone (i.e., the H‐only condition), a linear mixed‐effects (LME) model was applied to the scores of behavioral tests, with condition (LH vs. H‐only) as a fixed variable, and lesson, group, and student identities as random variables, while age and socioeconomic status (SES) as covariates. For the dependent variable, we subtracted the scores of pre‐test from that of lecturing‐test to index learning outcomes in the LH and H‐only conditions respectively. As expected, the results showed a significant main effect of condition (*F*_(1, 17.6)_ = 5.043, *p* = 0.038, *η* ^2^ = 0.062), with better learning outcomes in the LH condition than in the H‐only condition (*t*_(18.1)_ = 2.208, *p* = 0.040, Cohen's *d* = 0.520, 95% CI of the difference = [0.057, 0.971]; Figure 1E). These findings confirmed the previous pedagogical theory that blended teaching would produce better learning outcomes than did lecturing alone, due to additional involvement of recalling.

### 2.3 Distinct Brain Underpinnings for Different Styles of Classroom Teaching

**Figure 2:**
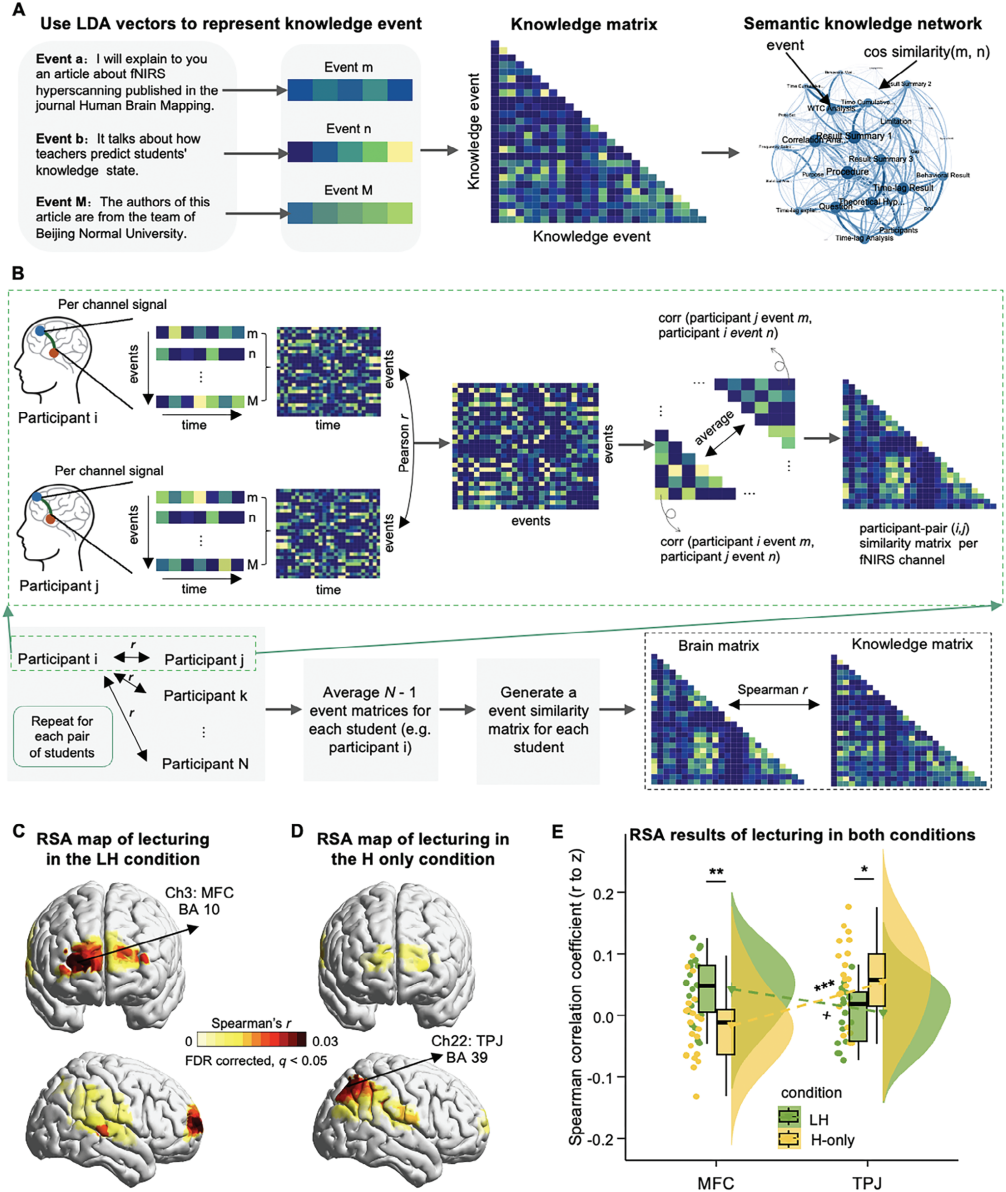
The pipeline and results of the representational similarity analysis (RSA) during the lecturing phase of both conditions. A) Creating the matrix of knowledge structure and the network of knowledge semantics. Each lecture's knowledge taught by the teacher was segmented into events with minimal conceptual unit. The semantic features of each event were transformed into sentence embedding vectors using the latent Dirichlet allocation (LDA) model. Cosine similarity was computed between each pair of knowledge events, thus generating a matrix of knowledge structure. A network of knowledge semantics was defined as a network whose nodes are knowledge events and the edge weights are the semantic similarity between events. B) An illustration of the RSA procedure. For example, for a pair of participants *i* and *j*, the preprocessed fNIRS signal corresponding to each knowledge event was extracted, and each participant generated a matrix (time × events) per channel. Next, an inter‐subject Pearson correlation method quantified the temporal correlations between the fNIRS signals corresponding to each pair of knowledge events, generating an event matrix (events × events) for this participant pair (*i*, *j*). We took the mean of this event matrix and its transpose to calculate the similarity matrix symmetric (i.e., similarity between events *m* and *n* across participants *i* and *j* = mean of corr (participant *i* event *m*, participant *j* event *n*) and corr (participant *j* event *m*, participant *i* event *n*)), and generating a brain similarity matrix for participant pair (*i*, *j*) per channel. The above analytic procedure was repeated across all possible pairs of students. For each student, *N –* 1 brain similarity matrix with the size of *M* × *M* would be generated, where *N* is the total number of students and *M* is the number of events within the knowledge. And then, the *N* − 1 event matrices were averaged and generated a single brain matrix per channel per student. Finally, Spearman correlation was calculated between the knowledge matrix and the brain activity matrix, and the correlation coefficients were averaged across students within each fNIRS channel. C) RSA results during the lecturing phase in the LH condition. The statistical map showed that knowledge was significantly represented in the MFC covered by CH3. D) RSA results during the lecturing phase in the H‐only condition. The significant representation of knowledge was found in the TPJ covered by CH22. E) Results of the condition × brain region ANCOVA during the lecturing phase using the LME method.

To investigate how students learned the knowledge through blended teaching, we performed representational similarity analyses (RSA, **Figure** **2**) between the knowledge taught by the teacher and the brain activity of students during the same lecturing phase of both the LH and H‐only conditions.^[^ ^50^ ^]^ We then compared the strength of knowledge representation between two conditions. It is worth noting again that, by employing different teaching materials and assigning different teachers to the two conditions, any potential carry‐over effects had been minimized, while the distinct contributions of recalling to the LH condition should be identified.

Specifically, four additional participants who did not participate in the teaching experiment were recruited to manually segment the knowledge into events with a minimal size of the conceptual unit (in total, 26 events in the LH condition and 27 events in the H‐only condition). The inter‐rater reliability was quantified using intra‐class coefficients (ICC), which reached a high level of 0.844. Vectorized semantic features of each knowledge event were obtained using the latent Dirichlet allocation (LDA) model.^[^ ^51^ ^]^ LDA is a widely used model in topic modelling to describe the distribution of latent semantic topics in text. These topics are represented by multiple distributions of vocabulary. Previous studies have frequently used LDA to quantitatively characterize text features and explore the semantic representations in the brain.^[^ ^52^ ^]^ Then, cosine similarity was calculated between each pair of events, generating a knowledge matrix (Figure 2A). To obtain the matrix of brain activity, the preprocessed fNIRS signal corresponding to each knowledge event was extracted. An inter‐subject Pearson correlation method was used to quantify the temporal correlations between the fNIRS signals (Figure 2B; see Experimental Section), to avoid the potential aliasing effect of autocorrelation in the hemodynamic signals within the same subject. Finally, Spearman correlation was calculated between the knowledge matrix and brain activity matrix (Figure 2B). It was worth noting that this correlation was conducted within each student, thus allowing us to detect the cortical representation of knowledge structure even when the knowledge had been constructed in different ways between one another. The correlation coefficients were averaged across students within each fNIRS channel. A permutation test was used to determine the statistical significance of the averaged correlation coefficient by randomly shuffling the time points of fNIRS signals for each student (see Experimental Section). The multiple comparison issue, due to the large number of fNIRS channels, was corrected using the false discovery rate (FDR) method (*q* < 0.05).

The results of the LH condition showed that the knowledge structure was significantly represented in the middle frontal cortex (MFC) covered by CH3 (*r* = 0.030, *p* = 0.030, one‐tailed permutation test; Figure 2C). To validate this result and test the consistency between the two lessons (13 and eight students in each lesson), the RSA was conducted separately for the two lessons. The results were well replicated in both lessons (lesson 1: CH 8: *r* = 0.038, *p* = 0.045; lesson 2: CH 6: *r* = 0.050, *p* = 0.019; one‐tailed permutation test; Figure S1C,D, Supporting Information). The same analytic procedures were applied to the H‐only condition (FDR corrected, *q* < 0.05). However, significant representation of knowledge structure was found in the TPJ covered by CH22 (*r* = 0.035, *p* = 0.016, one‐tailed permutation test; Figure 2D). Again, this result was consistently replicated across both lessons (lesson 1: CH 22: *r* = 0.058, *p* = 0.008; lesson 2: CH 21: *r* = 0.048, *p* = 0.042; one‐tailed permutation test; Figure S2A,B, Supporting Information).

Next, direct comparison between conditions and brain regions were conducted using the LME method, with condition and brain region as fixed variables, the identities of lesson, group, and student as random variables, while age and SES as covariates. The results did not show a significant main effect of either condition (*F*_(1, 77)_ = 0.147, *p* = 0.703, *η* ^2^ = 0.001) or brain region (*F*_(1, 77)_ = 1.856, *p* = 0.177, *η* ^2^ = 0.018). However, we found a significant interaction effect between condition and brain region (*F*_(1, 77)_ = 20.234, *p* < 0.001, *η* ^2^ = 0.195; Figure 2E). Further pairwise comparisons with Bonferroni correction showed significantly stronger representation of knowledge structure in the MFC of the LH condition than the H‐only condition (*t*_(58.6)_ = 3.435, *p* = 0.005, Cohen's *d* = 1.042, 95% CI of the difference = [0.423, 1.661]; Figure 2E). On the contrary, the TPJ showed stronger effect in the H‐only condition than in the LH condition (*t*_(58.6)_ = 2.896, *p* = 0.021, Cohen's *d* = 0.879, 95% CI of the difference = [0.249, 1.510]; Figure 2E). The other direction of pairwise comparisons showed marginally stronger representation of knowledge structure in the MFC than TPJ in the LH condition (*t*_(58.4)_ = 2.217, *p* = 0.092, Cohen's *d* = 0.670, 95% CI of the difference = [0.046, 1.293]; Figure 2E), but a significantly reversed pattern was found in the H‐only condition (*t*_(58.4)_ = 4.144, *p* < 0.001, Cohen's *d* = 1.252, 95% CI of the difference = [0.627, 1.876]; Figure 2E).

Together, these findings suggested that the brain underpinnings differed between styles of classroom teaching even during the same lecturing phase.

### 2.4 Testing the Global–Local Processing Hypotheses of Knowledge Construction during the Lecturing Phase

**Figure 3:**
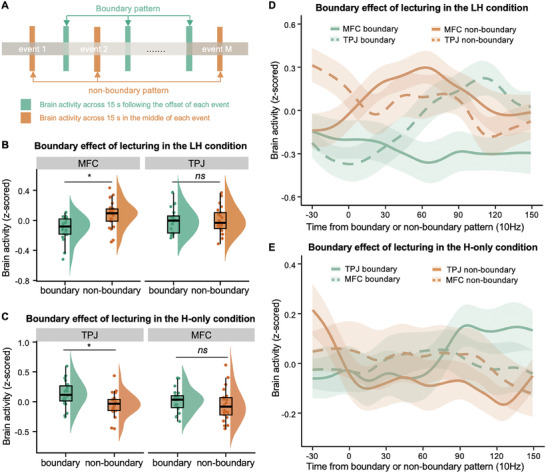
The analytic procedure and the results of boundary effect during the lecturing phase of both conditions. A) Schematic demonstration of the analysis. For each student, the boundary pattern was created by averaging the activation pattern across 15 s following the offset of an event (green bars), while the non‐boundary pattern was defined as the mean brain activity averaged across 15 s in the middle of an event (orange bars). Then, we compared the difference between boundary and non‐boundary patterns bra to index the strength of the boundary effect. B) The results of the boundary effect in the MFC and TPJ during the lecturing phase of the LH condition. An opposite pattern of the boundary effect was found in the MFC, with the boundary pattern being significantly lower than non‐boundary pattern. No boundary effect was found in the TPJ. C) The results of the boundary effect in the TPJ and MFC during the lecturing phase of the H‐only condition. A significant boundary effect was found in the TPJ, but none was found in the MFC. D,E) Time courses of the oxy‐hemoglobin (HbO) response were averaged across knowledge events in the LH condition (D) and the H‐only condition (E). It shows significant non‐boundary pattern than boundary pattern in the MFC of the LH condition only. Solid lines and dotted lines show responses of the MFC and TPJ, respectively. The green lines correspond to the boundary pattern, while the orange lines correspond to the non‐boundary pattern. For each line, the time courses were averaged first across knowledge events within each teaching phase, and then across participants. Shaded areas indicate the standard error of the mean across participants.

First, to determine whether the MFC and TPJ had processed the local attributes of knowledge structure during the lecturing phase in the LH condition, that is, segmenting the knowledge events in the same way as that of the original knowledge events or in a different way because of knowledge construction, we tested the boundary effect of knowledge events. Specifically, previous studies on the processing of naturalistic stimuli have shown that in higher‐order brain regions that represent the high‐level information of naturalistic stimuli, brain activity will automatically segment the stimuli into events.^[^ ^53^, ^54^, ^55^, ^56^ ^]^ The process of neural segmentation will result in higher neural responses at the offset of segments than at the middle of segments,^[^ ^57^, ^58^, ^59^ ^]^ a phenomenon referred to as the boundary effect. Here, we tested whether the brain representation of knowledge events would show a significant boundary effect during the lecturing phase of the LH condition. We assumed that, if the MFC or TPJ represented knowledge events without construction, it would segment the knowledge into events in the same way as the original segmentation rated by human participants, showing a significant boundary effect (i.e., the boundary pattern significantly higher than non‐boundary pattern); otherwise, they would construct the knowledge in a different way from the original segmentation, and no or a significantly reversed boundary effect (i.e., non‐boundary > boundary) would be found. Following previous studies,^[^ ^58^ ^]^ the boundary pattern was defined as the mean brain activity averaged across 15 s following the offset of an event, while the non‐boundary pattern was defined as the mean brain activity averaged across 15 s in the middle of an event (**Figure** **3**A). The time range of 15 s was selected because previous studies demonstrated that this duration captured most of boundary‐related signals, allowing for a more comprehensive assessment of brain activity patterns associated with event boundaries.^[^ ^58^, ^60^ ^]^ The difference between boundary and non‐boundary patterns was used to index the strength of the boundary effect.

In the LH condition, the results showed a significantly reversed boundary effect, with the non‐boundary pattern being significantly higher than the boundary pattern (paired sample *t*‐test: *t*_(20)_ = −2.854, *p* = 0.010, Cohen's *d* = −0.623, 95% CI of the difference = [−1.085, −0.148]; Figure 3B,D). Further analysis of individual knowledge events revealed that 25% of knowledge events showed this reversed pattern. These findings suggested that students’ MFC might have re‐segmented and constructed the knowledge events based on their prior experience and thus resulted in a shift in the positions of event boundaries. However, no such results were found in the TPJ of the LH condition (*p* > 0.05; Figure 3B,D).

The same procedure was applied to the TPJ and MFC of the H‐only condition. Different from the MFC in the LH condition, significant boundary effect was found in the TPJ, that is, higher boundary pattern than non‐boundary pattern (paired two‐sample *t*‐test: *t*_(20)_ = 2.612, *p* = 0.017, Cohen's *d* = 0.570, 95% CI of the difference = [0.102, 1.026]; Figure 3C,E), but none was found in the MFC (Figure 3C,E). Thus, it seemed that students’ TPJ represented the knowledge in the same way as that of the original knowledge structure in the H‐only condition, while the MFC constructed the knowledge structure through processing the local attributes of knowledge structure in the LH condition.

**Figure 4:**
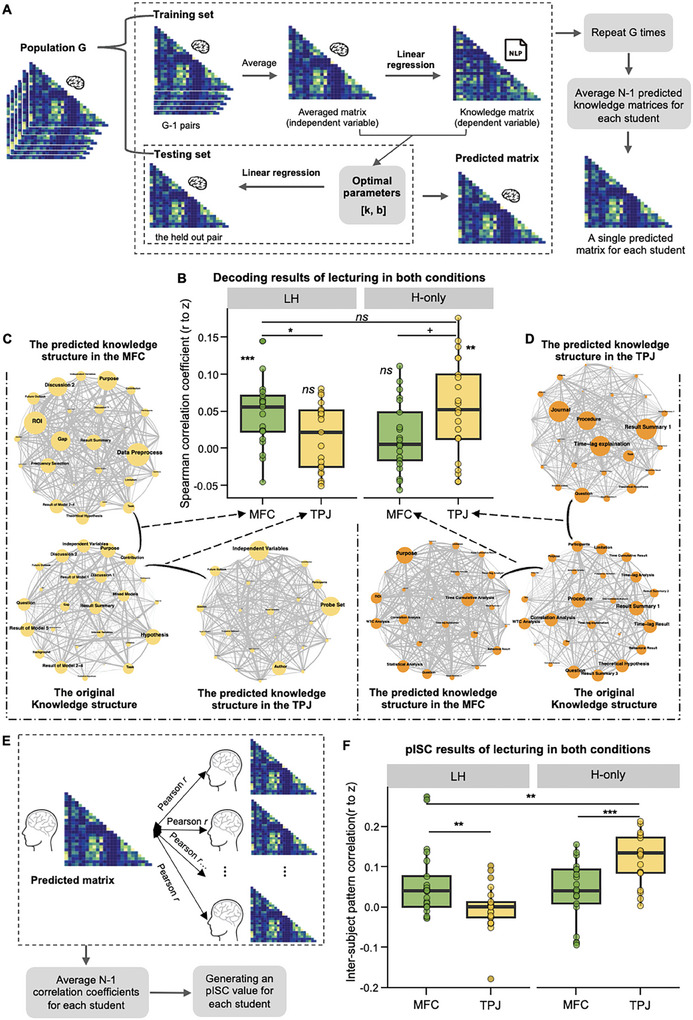
The pipeline of the decoding analysis and its results during the lecturing phase of both conditions. A) Schematic demonstration of the decoding analysis. It was conducted by a leave‐one‐dyad–out procedure. Specifically, 1) Using the RSA procedure (see Figure 2B), we obtained the brain matrix for each pair of students via the inter‐subject Pearson correlation. These matrices formed a population *G*. 2) *G* was divided into a training set (*G* − 1 pairs) and a testing set (1 pair). The training set matrices were averaged to train the regression model, with brain activity as the independent variable and the original knowledge matrix as the dependent variable. 3) The testing set (the held‐out pair) was then used to generate a predicted knowledge matrix. 4) Steps 2 and 3 were repeated *G* times. Each student's *N −* 1 predicted knowledge matrices were averaged into a single predicted knowledge matrix. B) Decoding results of the lecturing phase in both conditions. The results showed a significant similarity between the predicted and original knowledge structure in the MFC of the LH condition and in the TPJ of the H‐only condition, and no significant difference between the similarity obtained from the MFC in the LH condition and that from the TPJ in the H‐only condition. C,D) The network visualization of the original and the predicted knowledge structures from the MFC and TPJ in the LH (yellow networks) and H‐only condition (orange networks). The node size is proportional to the degree (i.e., the mean of the similarity value of all edges connected to the node in the network). Edge thickness is proportional to the edge weights. Since each student had her/his distinct knowledge structure as predicted, we chose to present the predicted knowledge structure of one representative student in the figure, and this approach was also applied to the subsequent network visualization figures. E) The pISC analysis of decoding results. We computed the Pearson correlation between the predicted knowledge matrix of each student and that of all other students, generating *N −* 1 correlation coefficients. The coefficients were further averaged and obtained an pISC value for each student. F) pISC results. The results showed significantly larger individual variance (i.e., lower pISC) in the MFC of the LH condition than in the TPJ of the H‐only condition.

Second, to test whether the global attributes of knowledge structure had been processed during the lecturing phase in the LH condition, we employed a brain decoding procedure. Specifically, 1) we obtained the brain activity matrix for each possible pair of students via the inter‐subject Pearson correlation (Figure 2B). These matrices formed a population *G*. 2) *G* was divided into a training set (*G* − 1 pairs) and a testing set (1 pair). The matrices in the training set were averaged to train the regression model, with brain activity as the independent variable and the original knowledge matrix as the dependent variable. 3) The testing set (the held‐out pair) was then used to generate a predicted knowledge matrix. 4) Steps 2–3 were repeated *G* times. Each student's *N −* 1 predicted knowledge matrices were averaged into a single predicted knowledge matrix (**Figure** **4**A). 5) Spearman correlation between the predicted and original knowledge matrices was calculated and converted to Fisher *z* for normalization. 6) To evaluate the performance of knowledge construction, a one‐sample *t*‐test was conducted on the *z*‐values across students against zero.

The results showed a significant similarity between the predicted and original knowledge structures in the MFC of the LH condition (*t*_(20)_ = 4.693, *p* < 0.001, Cohen's *d* = 1.024, 95% CI of the difference = [0.484, 1.547]; Figure 4B,C). The same analyses were conducted on the TPJ during the lecturing phase of the H‐only condition and produced a similar result, namely, a significant similarity between the predicted and original knowledge structures (*t*_(20)_ = 3.693, *p* = 0.001, Cohen's *d* = 0.806, 95% CI of the difference = [0.304, 1.293]; Figure 4B,D). Moreover, no significant difference was found between the similarity obtained from the MFC in the LH condition and that from the TPJ in the H‐only condition (paired sample *t*‐test: *t*_(20)_ = 0.228, *p* = 0.822, Cohen's *d* = 0.050, 95% CI of the difference = [−0.379, 0.477]; Figure 4B–D).

Based on these findings, we reasoned that, although the MFC representation of knowledge structure was similar to the original knowledge structure in the overall network pattern, the MFC representation might have a larger individual variance in the LH condition than that of the TPJ in H‐only condition due to the impact of prior personal experience as well as different processing of local attributes of knowledge structure on knowledge construction. To test this possibility, an inter‐subject pattern correlation (pISC) analysis was conducted in both conditions (Figure 4E). It highlighted individual consistency by assessing the pattern similarity of predicted knowledge structure across students, thus providing insight into how differently knowledge structure was represented across individuals in each condition (i.e., larger individual variance corresponding to lower pISC value).^[^ ^19^ ^]^ As expected, the results showed significantly larger individual variance in the MFC of the LH condition than in the TPJ of the H‐only condition (paired sample *t*‐test: *t*_(20)_ = 2.914, *p* = 0.009, Cohen's *d* = 0.636, 95% CI of the difference = [0.159, 1.100]; Figure 4F). Moreover, significantly lower individual variance was found in the TPJ than MFC of the LH condition (*t*_(20)_ = 3.428, *p* = 0.003, Cohen's *d* = 0.748, 95% CI of the difference = [0.255, 1.227]) and vice versa for the H‐only condition (*t*_(20)_ = 4.134, *p* < 0.001, Cohen's *d* = 0.902, 95% CI of the difference = [0.384, 1.404]; Figure 4F).

Taken together, these findings supported both the global and local processing hypotheses of knowledge construction during blended teaching, but suggested a prioritized processing of local attributes during the lecturing phase. They also suggested a functional dissociation between the TPJ and MFC, with the former only representing the original knowledge structure, whereas the latter constructing knowledge into a different structure from the original one because of blended teaching.

### 2.5 The Knowledge Construction in the MFC might have Started before the Lecturing Phase in the LH Condition

**Figure 5:**
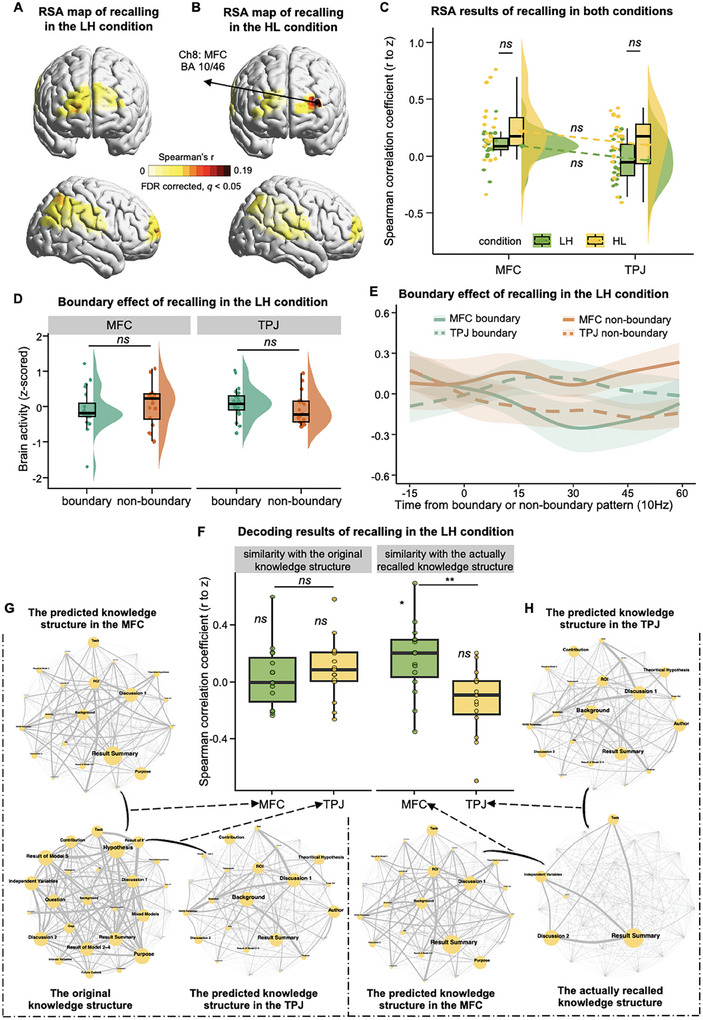
The results during the recalling phase of the LH condition. A) RSA results during the recalling phase in the LH condition. The RSA map showed the largest *r* values in the MFC (CH3) across all CHs, but it did not survive the FDR correction at the *p* < 0.05 level. B) RSA results during the recalling phase in the HL condition. The significant representation of knowledge was found in the MFC covered by CH8. C) Results of the condition × brain region ANCOVA during the recalling phase using LME. There was no significant effect of interaction between conditions and brain regions. D) The results of boundary effect in the MFC and TPJ during the recalling phase of the LH condition. The boundary effect was not found either in the MFC or TPJ. E) In the LH condition, time courses of the HbO responses averaged across events in the MFC and TPJ during the recalling phase. The format and structure of the data presentation are the same as those in Figure 3D. Note that due to the limited duration of the recalled knowledge events, the duration for boundary and non‐boundary patterns was set to 6 s. F) Decoding results during the recalling phase in the LH condition. There was no significant similarity between the predicted knowledge structure generated from the MFC activity and the original knowledge structure. However, a significant similarity was observed across students between the predicted knowledge structure based on the MFC activity and the knowledge structure actually recalled by students during the recalling phase. G) The network visualization of the original knowledge structure and that decoded from the MFC and TPJ during the recalling phase of the LH condition. H) The network visualization of the knowledge structure actually recalled by a student and the predicted knowledge structure from the MFC and TPJ during the recalling phase of the LH condition.

To test whether knowledge construction in the MFC of the LH condition was specific to the lecturing phase, we conducted the RSA during the recalling phase immediately before the lecturing phase. To build the similarity matrix based on the knowledge recalled, verbal reports of students were transcribed into text, segmented, and converted into LDA vectors. The similarity matrix was generated for each student by calculating the Cosine similarity between each pair of knowledge events. The matrices were then averaged across all students, generating a group‐averaged knowledge matrix. It should be noted that due to the fact that different students recalled different subsets of events, the number of students averaged varied across event pairs, and might be zero for some events. Brain activity matrix was built in the same way as above for each individual student (Figure 2B). Finally, Spearman correlation was conducted between the two matrices for each student and the correlation coefficients were then averaged across all students to create a single value per channel. The permutation test was used to determine the statistical significance (see Experimental Section). The results showed the largest *r* values in the MFC (CH 3; *r* = 0.093, *p* = 0.066, one‐tailed permutation test) across all CHs, though it did not survive the FDR correction at the *p* < 0.05 level (**Figure** **5**A).

Next, the boundary effect and decoding analyses were conducted to examine the global–local hypotheses. It should be noted that the duration used for the calculation of boundary and non‐boundary patterns was set to 6 s due to the limited duration of knowledge events recalled by students. The results did not show any significant boundary effects in the MFC, nor was there a significantly reversed pattern (paired sample *t*‐test: *t*_(19)_ = −0.686, *p* = 0.501, Cohen's *d* = −0.153, 95% CI of the difference = [−0.592, 0.290]; Note that one student did not correctly recall any knowledge events, thus resulting in a value of 19 for the degree of freedom; Figure 5D,E), suggesting a weak processing of local attributes of knowledge structure during the recalling phase.

Moreover, the brain‐decoding approach did not find a significant similarity between the predicted knowledge structure based on the MFC activity and the original knowledge structure, (one sample *t*‐test: *t*_(14)_ = 0.901, *p* = 0.385, Cohen's *d* = 0.250, 95% CI of the difference = [−0.308, 0.798]; Note that six students recalled fewer than three knowledge events, resulting in a degree of freedom of 14; Figure 5F,G), but a significant similarity was observed between the predicted knowledge structure generated by the MFC activity and the knowledge structure actually recalled by students during the recalling phase (*t*_(14)_ = 2.461, *p* = 0.030, Cohen's *d* = 0.683, 95% CI of the difference = [0.064, 1.278]; Figure 5F,H).

Thus, it seemed that, at this step, MFC construction might have prioritized the global rather than the local attributes of knowledge structure. Moreover, they also suggested that knowledge construction might have started before the lecturing phase but was significantly facilitated by it, transitioning from prioritizing the processing of the global to the local attributes of knowledge structure.

### 2.6 The Order Effect of Blended Teaching

To test whether the sequence of blended teaching affected learning outcomes, students were asked to freely recall the knowledge in the same way as that in the LH condition immediately after teacher's lecturing, generating an HL condition. Students were tested after their recalling (recalling‐test). We examined the order effect between LH and HL conditions by comparing learning outcomes of the two conditions using the LME method. The dependent variable was calculated by subtracting the pre‐test score from that of the lecturing‐test in the LH condition or that of the recalling‐test in the HL condition (Figure 1D). The results did not show a significant main effect of condition (*F*_(1, 38)_ = 1.086, *p* = 0.304, *η* ^2^ = 0.026; Figure 1F), suggesting that, at the behavioral level, there was no order effect associated with the sequence of blended teaching.

Next, we investigated the order effect at the brain level using RSA. Specifically, we examined the recalling phase of the HL condition (i.e., recalling immediately after the lecturing, FDR corrected, *q* < 0.05). As expected, we found a significant representation of knowledge structure in the MFC (CH 8; *r* = 0.191, *p* = 0.009, one‐tailed permutation test; Figure 5B) but not in the TPJ (CH 22). Moreover, direct comparisons between conditions and brain regions were conducted using the LME method, with condition and brain region as fixed variables, and the identities of lesson, group, and student as random variables, while age and SES as covariates. There was a significant main effect of condition (*F*_(1, 70.2)_ = 7.765, *p* = 0.007, *η* ^2^ = 0.085), with stronger representation of knowledge structure during the recalling phase of the HL condition than that of the LH condition (Figure S3A, Supporting Information). There was also a significant main effect of brain region (*F*_(1, 68.4)_ = 7.279, *p* = 0.009, *η* ^2^ = 0.077), with stronger representation of knowledge structure in the MFC than TPJ during the recalling phase (Figure S3B, Supporting Information). However, no significant interaction effect was found between condition and brain region (*F*_(1, 68.4)_ = 0.003, *p* = 0.958, *η* ^2^ = 0.000; Figure 5C).

**Figure 6:**
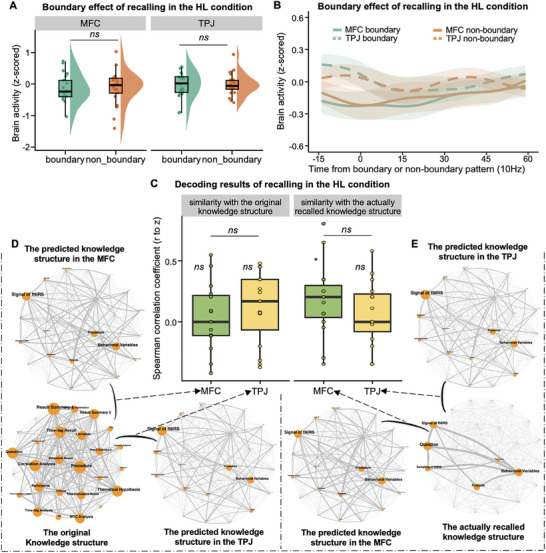
The results during the recalling phase of the HL conditions and mutual facilitation of the two teaching phases. A) The results of boundary effect in MFC during the recalling phase in the HL condition. There is no significant boundary effect either in the MFC or TPJ. B) In the HL condition, time courses of the HbO responses averaged across events in the MFC and TPJ during the recalling phase. The format and structure of the data presentation are the same as those in Figures 3D and 5E. C) Decoding results during the recalling phase in the HL condition. There was no significant similarity between the predicted knowledge structure in the MFC and the original knowledge structure, but a significant correlation was found with the knowledge structure each student actually recalled. D) The network visualization of the original and the predicted knowledge structures from the MFC and TPJ during the recalling phase of the HL condition. E) The network visualization of the knowledge structure actually recalled by a student and the predicted knowledge structure from the MFC and TPJ during the recalling phase of the HL condition.

Additionally, the MFC activity during the recalling phase in the HL condition did not show significant boundary effect, nor was there a significantly reversed boundary effect (paired sample *t*‐test: *t*_(20)_ = −0.175, *p* = 0.863, Cohen's *d* = −0.038, 95% CI of the difference = [−0.466, 0.390]; **Figure** **6**A,B). Most importantly, the brain‐decoding procedure did not show significant similarity between the predicted knowledge structure generated by the MFC activity and the original knowledge structure (Figure 6C,D). On the contrary, we found a significant correlation between the predicted knowledge structure generated by the MFC activity and the knowledge structure each student actually recalled (one sample *t*‐test: *t*_(14)_ = 2.217, *p* = 0.044, Cohen's *d* = 0.572, 95% CI of the difference = [0.016, 1.112]; Figure 6C,E). The same analytic procedure was repeated on the TPJ during the recalling phase, but none was found (Figure 6D,E). These findings replicated the findings of the recalling phase in the LH condition; that is, knowledge would be effectively constructed in the MFC rather than the TPJ, and at this step, MFC construction might have prioritized the processing of the global attributes rather than the local attributes of knowledge structure.

### 2.7 Mutual Facilitation of the Two Teaching Phases

**Figure 7:**
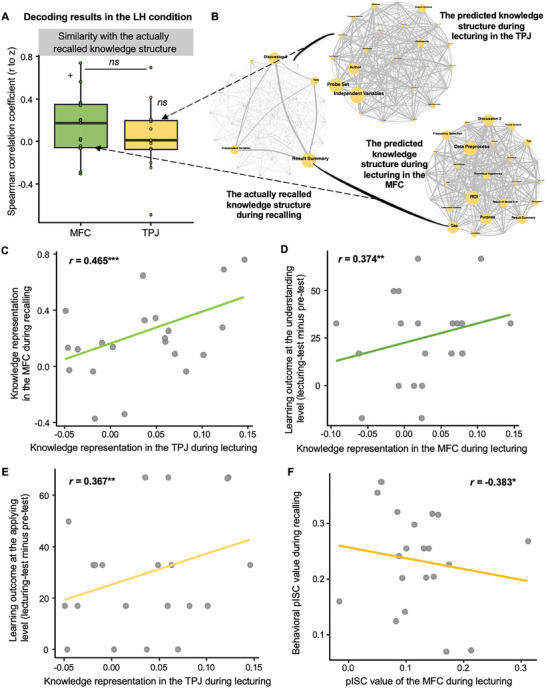
Mutual facilitation of the two teaching phases and correlation results between MFC's knowledge construction and learning outcomes. A) In the LH condition, the decoding results showed a marginally significant similarity between the predicted knowledge structure based on the MFC activity during the lecturing phase and the actually recalled knowledge structure during the recalling phase. B) The network visualization of the actually recalled knowledge structure during the recalling phase and the predicted knowledge structures from the MFC and TPJ during the lecturing phase of the LH condition. C) The correlation results showed a significantly positive correlation between the representation of knowledge in the TPJ during the lecturing phase of the H‐only condition and that of the MFC during the recalling phase of the HL condition. D) In the LH condition, a significant correlation was found in the MFC during the lecturing phase with the learning outcome at the understanding level (i.e., lecturing‐test‐pre‐test). E) In the HL condition, a significant correlation was found in the TPJ during the lecturing phase with the learning outcome at the applying level (i.e., lecturing‐test‐pre–test). F) The correlation results revealed a significantly negative correlation between the pISC value of recalling performance and the pISC value of the MFC during the lecturing phase in the LH condition.

To test the relation between the two teaching phases, we calculated correlation between the predicted knowledge structure generated by the MFC activity during the lecturing phase and the knowledge structure actually recalled by students during the recalling phase of the LH condition, and obtained a marginally significant correlation (*t*_(14)_ = 1.911, *p* = 0.077, Cohen's *d* = 0.493, 95% CI of the difference = [−0.052, 1.023]; **Figure** **7**A,B). This finding suggested a facilitation effect the prior processing of the global attributes on later processing of local attributes of the knowledge structure.

We also calculated the correlation between the representation of knowledge in the TPJ during the lecturing phase and that of the MFC during the recalling phase immediately after lecturing in the HL condition, and obtained a significantly positive correlation (*r* = 0.465, *p* < 0.001). This finding also indicated that the better the brain representation of the original knowledge structure during lecturing, the better the subsequent knowledge construction during recalling (Figure 7C). These findings together suggested a mutual facilitation of the two teaching phases.

### 2.8 Validation on the RSA Results through Random Permutation

To further validate the brain underpinnings of knowledge representation and construction during high‐ and low‐structured teaching phases in both conditions, we generated random data for the two conditions by shuffling the experimental data. Specifically, we combined the data from the high‐structured teaching phases in both conditions to form a unified database. Next, we randomly selected half of the data from this database for the LH condition, while the other half of the dataset was used for the HL condition. Subsequently, these random datasets underwent RSA analysis separately for high‐ and low‐structured teaching phases again. As expected, the results did not show any significant neural pattern either in the high‐structured teaching phase or in the low‐structured teaching phase in both conditions (Figure S4, Supporting Information).

### 2.9 Correlation between MFC's Knowledge Construction and Learning Outcomes

The above results highlighted the pivotal role of the MFC in knowledge construction. We further examined whether the MFC's activity correlated to each level of students’ learning outcomes (i.e., Remembering, Understanding, and Applying) after all teaching phases using a partial Pearson correlation approach (one‐tailed, *p* < 0.05), while students’ age and SES were controlled. In the LH condition, the results showed a significant correlation between the strength of the MFC's representation during the lecturing phase (*r* = 0.374, *p =* 0.008) with the learning outcome at the understanding level (i.e., lecturing‐test minus pre‐test; Figure 7D). None was found between the strength of the MFC's representation during the recalling phase, or the strength of the TPJ's representation during the lecturing phase with learning outcomes (i.e., recalling‐test minus pre‐test) at any levels in the HL condition (*p*s > 0.05).

Further analyses were conducted between the strength of brain representations and learning outcomes in each phase. The results revealed that, in the HL condition, the TPJ's neural representation during the lecturing phase showed a significant positive correlation (*r* = 0.367, *p* = 0.008) with the learning outcome at the applying level (i.e., lecturing‐test minus pre‐test; Figure 7E). However, we did not find significant correlations between the MFC's representation of knowledge and learning outcomes at any levels during the recalling phase in the HL condition, nor were there any significant correlations in the LH conditions (*p*s > 0.05).

To delineate how the MFC activity was associated with learning outcomes, a pISC analysis was conducted in the LH condition. Specifically, we employed binary encoding (0,1) procedure, where correctly recalled knowledge events were coded as 1 and other segments as 0, obtaining a performance vector for each student. We computed the similarity between each student and all of other students on the recalling performance vector using the Jaccard similarity method and averaged the similarity across all potential correlations, deriving a behavioral pISC value for each student, which reflected the consistency (approaching 1) or inconsistency (approaching 0) of the recalling performance among students. The behavioral pISC value was correlated to the pISC value of the MFC during lecturing using a Pearson correlation method. A significantly negative correlation was observed in the LH condition (*r* = −0.383, *p* = 0.043; Figure 7F). This finding suggested that the more the knowledge was constructed differently among students, the better the student's learning performance.

### 2.10 Teacher was Involved in Student's Knowledge Construction through Teacher–Student Neural Synchronization

**Figure 8:**
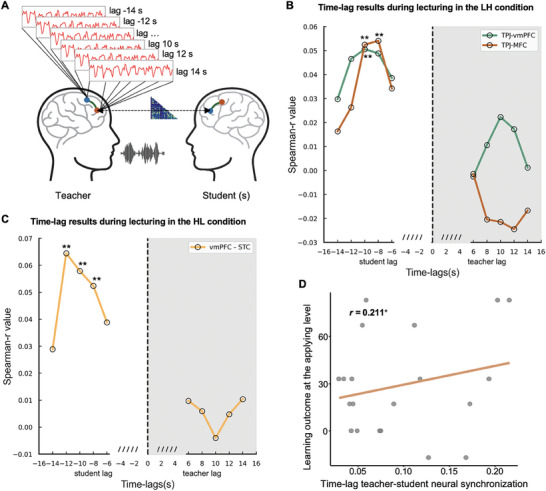
The results of teacher–student neural synchronization and behavioral correlation. A) The analysis of teacher–student neural synchronization. Note that the teacher's brain activity was shifted forward or backward by 6–14 s (step = 2 s) relative to the students’ brain activity. B) The results of time‐lag teacher–student neural synchronization during the lecturing phase of the LH condition. The results showed that the teacher's brain activity in the right TPJ (CH17 and 19) positively correlated with the student's brain activity in the MFC (CH 8 and CH 5) when the teacher's brain activity preceded that of the student by 8–10 s, with a peak correlation at a time lag of 8 s. C) The results of time‐lag teacher–student neural synchronization during lecturing in the HL condition. The teacher's brain activity in the MFC (CH 9) positively correlated with the student's brain activity in the superior temporal cortex (STC, CH 13) when the teacher's brain activity preceded that of the student participants from 8 to 12 s. This effect reached a peak when the time lag was 12 s. D) The result showed a marginally positive correlation between the time‐lag teacher–student neural synchronization and the learning outcome at the applying level in the LH condition.

To test how the teacher was associated with student's knowledge construction, we examined whether the teacher's brain representation of knowledge structure predicted students’ brain representations of knowledge structure by calculating RSA between the teacher's brain activity matrix and that of students for all possible CH combinations during the same lecturing phase of both conditions (i.e., 22 × 22 = 484, see Experimental Section; **Figure** **8**A). Moreover, previous studies have demonstrated a specific association between the time‐lag neural synchronization and teaching process, that is, only when the teacher's brain activity preceded that of students, the teacher–student neural synchronization would correlate with the learning outcomes.^[^ ^42^, ^45^ ^]^ Thus, time‐lags from 6 to 14 s were added to the computation of neural synchronization based on previous studies. The statistical significance was determined by a permutation test in which the time series of fNIRS were randomly shuffled. The results were corrected for multiple comparisons across all CH pairs (FDR, *q* < 0.05).

The results of the LH condition showed that the teacher's brain activity in the right TPJ (CH 17 and 19) positively correlated with the student's brain activity in the MFC (CH 8 and 5) when the teacher's brain activity preceded that of the student by 8–10 s, with a peak correlation at a time lag of 8 s (from teacher's TPJ to students’ MFC; *r* = 0.054, *p =* 0.001, FDR corrected; Figure 8B). No other significant results were found when the student's brain activity preceded that of the teacher per time‐lag per CH pair (*ps* > 0.05, FDR corrected; Figure 8B). In the HL condition, we found that teacher's brain activity in the MFC (CH 9) positively correlated with the student's brain activity in the superior temporal cortex (STC, CH 13) when the teacher's brain activity preceded that of the student from 8 to 12 s (*ps* < 0.01, FDR corrected; Figure 8C). This effect reached a peak when the time lag was 12 s (*r* = 0.064, *p =* 0.001, FDR corrected; Figure 8C). No significant results were found when the student's brain activity preceded that of the teacher per time‐lag per CH pair (*ps* > 0.05, FDR corrected; Figure 8C).

To further validate the association between teacher–student neural synchronization and learning outcomes, partial correlations were conducted between the time‐lag teacher–student brain similarity at the peak and students’ learning outcomes, while students’ age and SES were controlled. The results of the LH condition showed a marginally significant correlation between teacher–student neural synchronization and learning outcome (lecturing‐test‐pre–test) at the applying level (*r* = 0.211, *p* = 0.093; Figure 8D). In the HL condition, however, we did not observe any significant or marginally significant correlation.

Therefore, these findings indicated that a successful knowledge construction in the MFC of students was preceded by a directional projection of knowledge structure from the teachers’ TPJ. Moreover, the teachers’ positive contribution only occurred in blended teaching when lecturing was preceded by recalling, though the final learning outcomes did not significantly differ between the LH and HL conditions.

## 3 Discussion

In this study, we examined the neurocognitive mechanisms of real classroom teaching in facilitating learning outcomes. We demonstrated that blended teaching effectively facilitated students’ knowledge construction in the MFC, which significantly correlated with high‐level rather than low‐level learning outcomes. Below, we expand on the details.

First, in previous pedagogical theories on effective teaching, there is a well‐recognized perspective that the outcome of blended teaching is better than that of lecturing alone.^[^ ^14^, ^15^, ^61^, ^62^ ^]^ However, its underlying neurocognitive mechanisms remain elusive due to the gap between pedagogical speculation and empirical investigation among the educational, cognitive, and neuroscientific literature. According to knowledge construction theories in the science of learning, the purpose of classroom teaching is not only to facilitate the precise representation of the semantics of knowledge events in the brain, but also the construction of the relation between events.^[^ ^9^, ^63^, ^64^ ^]^ Therefore, we hypothesized that facilitating knowledge construction in the students’ brains is probably the neurocognitive mechanisms driving the positive effect of blended teaching. In our study, a low‐structured recalling phase was introduced prior to the lecture in the LH condition. This phase was designed to activate students’ prior knowledge and encourage them to retrieve relevant information, effectively creating a cognitive scaffold before engaging with new content. By doing so, the subsequent high‐structured lecturing phase in the LH condition built upon this activated prior knowledge, potentially leading to more effective integration of new knowledge and restructuring of knowledge events. Our results supported this hypothesis, showing that, during the same lecturing phase, knowledge was represented in the MFC during blended teaching but in the TPJ during lecturing alone. Moreover, our results indicated that the MFC segmented knowledge into events in a different way from the original segmentation rated by human participants, whereas the TPJ did not, suggesting a reorganization of the relations between knowledge events, that is, the construction of the intrinsic structure of knowledge in the MFC. Furthermore, the MFC's representation of the constructed knowledge showed greater individual variance in the LH condition than the TPJ in the H‐only condition. To our knowledge, this is the first evidence demonstrating the MFC's role in knowledge construction during real‐world classroom teaching. These findings also suggest that the MFC processed both the global and local attributes of the knowledge structure during construction.

Second, this finding supported the rapid cortical learning theory, which posits that a cortical learning system can quickly form memory engrams during learning within minutes to hours, and support the representation and construction of knowledge.^[^ ^32^, ^65^, ^66^ ^]^ Compared to the TPJ, previous studies have shown that the MFC is pivotal in integrating new information with existing knowledge, thereby facilitating the construction of coherent knowledge structures.^[^ ^67^, ^68^ ^]^ Specifically, during conceptual learning, neural representations in the lateral PFC (including MFC) can track the efficient mapping of stimuli to categories and build organized knowledge structures through selectively weighting relevant and irrelevant features.^[^ ^69^ ^]^ A recent study further found that students with better working memory performance showed more neural synchronization in the MFC during video learning.^[^ ^70^ ^]^ Additionally, a meta‐analysis illustrates that damage to the MFC disrupts the cognitive processes essential for integrating new information and creative thinking.^[^ ^71^ ^]^ Unlike previous studies conducted in the highly controlled laboratory or clinical context,^[^ ^31^, ^35^, ^72^, ^73^ ^]^ our study elucidated the significant role of the MFC in knowledge construction in the real‐world classroom context, providing novel insights into the function of MFC in classroom teaching and complementing the theoretical framework of the rapid cortical learning system.

Third, our results specifically showed that the MFC's construction of knowledge aligned with both the global and local processing hypotheses. The global–local processing theory suggests that knowledge construction is shaped by how students perceive the attributes of knowledge structure.^[^ ^26^ ^]^ When students focus on global attributes, they organize and integrate new information by understanding the broader knowledge framework. Conversely, local attributes involve mastering individual knowledge events and their relationships. The emphasis on these attributes can differ, influencing the approach to knowledge construction.^[^ ^27^ ^]^ In other words, constructing global attributes often leads to a different knowledge structure due to reorganization and disruption of the original structure. Conversely, focusing on local attributes tends to result in a knowledge structure similar to the original, as only the local attributes are processed without fundamentally altering the overall structure. In our study, we showed that, although the overall structure of knowledge decoded from the MFC was similar to the original one during the lecturing phase in the LH condition, the presence of a local boundary effect, as well as larger individual variance in representing the overall knowledge structure, suggested both global and local changes in the constructed knowledge in the MFC. Moreover, the result also suggested a prioritized processing of the local attributes during the lecturing phase, which supported the importance of local attribute perception in knowledge construction. This is consistent with the evidence that participants can adjust their attention focus according to the task requirements during the letters processing, becoming more efficient in processing local elements.^[^ ^74^ ^]^ Additionally, we clarified how blended teaching facilitated the MFC's knowledge construction, showing that this process also occurred during the recalling phase, either prior to or post the lecturing phase. These findings suggested a transition of knowledge construction from prioritizing the global to the local attributes of knowledge structure in the LH mode, but not in the HL mode of blended teaching. Thus, the LH mode appears to have a superiority effect over the HL mode in facilitating knowledge construction.

Fourth, our results additionally showed an active involvement of teachers in the MFC's function of learners. Specifically, we found that the teacher was involved in students’ knowledge construction through teacher–student neural synchronization during lecturing in the LH mode of blended teaching. The teacher's brain activity in the right TPJ positively correlated with the student's brain activity in the MFC when the former predicted that of the latter by 8–10 s. This result closely replicated our previous work,^[^ ^42^ ^]^ which showed that the brain activity of the teacher in the TPJ could predict the students’ knowledge state in the anterior superior temporal cortex up to 10 s in advance, allowing the teacher to formulate an appropriate representation of the knowledge for transmission to the student. According to the zone of proximal development theory,^[^ ^64^ ^]^ which suggests that teachers should dynamically predict students’ knowledge levels, identify their zone of proximal development, and create suitable knowledge representations to transmit,^[^ ^42^, ^75^ ^]^ our results provided additional empirical support and initial evidence in the classroom context for this theory in a classroom context. Additionally, in the H‐only condition, we also found teacher–student neural synchronization between the teachers’ MFC and the students’ STC, with a delay of 8–12 s. This pattern of interpersonal neural synchronization almost reversed the direction of neural synchronization in the LH condition. Moreover, no significant correlation was found between this interpersonal neural synchronization and learning outcomes, suggesting that lecturing alone or lecturing before recalling is not as effective as lecturing after recalling in facilitating knowledge construction. These findings suggested a particularly important role of teachers in students’ knowledge construction in the MFC during blended teaching.

There are several limitations to this study. First, to investigate the neurocognitive mechanisms of blended teaching in a real classroom, we selected a university's graduate course. Thus, only 24 university students who registered for this course participated in the study. The relatively small sample size may weaken the statistical power of our results. Particularly, in our study, the number of male participants was pretty low. Thus, it is important to replicate these findings with a larger sample and a more balanced gender distribution, as well as in studies exploring different learning materials or age populations. Second, we focused only on the short‐term effects of classroom teaching. Therefore, it remains unclear whether and how long the positive effect of blended teaching on knowledge construction will persist. Long‐term effects should be investigated in future studies. The third limitation of this study is that we only employed novice teachers with about one year of teaching experience. Indeed, teachers’ experience might have influence on our results.^[^ ^76^, ^77^ ^]^ Experienced teachers may excel at connecting new knowledge with students’ existing understanding, which could potentially reduce the differences observed between the LH and H‐only conditions. While we acknowledge this potential effect, we are concerned that experienced teachers may have larger individual variances in teaching capabilities. For example, some experienced teachers may excel at classroom teaching, while others may be skilled at creating a conductive learning environment. These individual differences may introduce additional unknown confounding factors. Therefore, in our study, we recruited novice teachers with ≈1 year of teaching experience to minimize the potential influence of these unknown confounding factors. Fourth, while fNIRS is suitable for studying classroom teaching and has potential for investigating the role of the rapid cortical learning system in knowledge construction, its limited spatial resolution and depth of penetration leave open the question of whether other brain regions, such as the hippocampus, are involved in classroom teaching.^[^ ^30^ ^]^ Concurrent fMRI‐fNIRS scanning may help resolve this dilemma. Finally, to balance the need to measure more brain regions and the difficulty in collecting data from many students in the real classroom, we only measured the TPJ on the right hemisphere. This arrangement may have overlooked other brain regions that also have a crucial role in classroom teaching. Therefore, we hope that our study will encourage more researchers to replicate and extend our findings in future to further advance our understanding on real classroom teaching.

In summary, our study provides empirical evidence for the neurocognitive mechanisms underlying the effectiveness of real classroom teaching in facilitating knowledge construction among students. We demonstrated that blended teaching promotes knowledge construction in the MFC with the processing of both global and local attributes, which significantly correlates with high‐level learning outcomes. This contrasts with traditional lecturing, where knowledge was represented as it is in the TPJ. Our findings highlight the critical role of the MFC in reorganizing the intrinsic structure of knowledge, supporting the rapid cortical learning theory. Additionally, teacher–student neural synchronization, particularly in the LH mode of blended teaching, emphasizes the active role of teachers in facilitating students’ knowledge construction by effectively identifying students’ zone of proximal development and delivering appropriate knowledge representations. Together, these findings challenge the boundaries of traditional science of learning by enriching our understanding of the cognitive and neural processes involved in real classroom teaching, and offer a novel perspective on optimizing educational practices through studies in basic cognitive neuroscience.

## 4 Experimental Section

### Participants

A power analysis was conducted using G*power (version 3.1.9.6).^[^ ^78^ ^]^ An effect size of *f* = 0.30 was used aiming for 0.80 power at 0.05 alpha error probability in the LME analysis.^[^ ^79^ ^]^ The power analysis indicated that 18 participants were required. This sample size also aligns with other studies in the fields of educational neuroscience.^[^ ^44^, ^80^ ^]^ Finally, twenty‐four postgraduate students were included (16 females; age: *M* = 25.476, SD = 2.926). They have taken five lessons in this course prior to this study, thus already having background knowledge about the principles of brain imaging, brain structure, and functions, but they have not learnt to read research papers with complete structures. Students in the two lessons did not significantly differ in their demographic characteristics (Table S2, Supporting Information). Four additional participants, majoring in brain imaging and acting as teaching assistants for the neuroimaging course, were assigned as teachers (females, age: *M* = 26.500, SD = 1.291). These participants were pre‐service teachers who had undergone 3 to 4 years of professional teacher training prior to the study and had at least 6 months of actual teaching experience. Their training ensured they were well‐prepared to deliver consistent teaching throughout the study. All participants were right‐handed,^[^ ^81^ ^]^ had normal hearing and normal or corrected‐to‐normal vision, and had no history of neurological diseases. Participants received monetary compensation for their time. The study protocol was reviewed and approved by the Institutional Review Board of the State Key Laboratory of Cognitive Neuroscience and Learning, Beijing Normal University, and all participants provided written informed consent.

### Materials and Procedures

Four scientific papers related to fNIRS hyperscanning were selected as the teaching materials.^[^ ^40^, ^42^, ^82^, ^83^ ^]^ The selection criteria required that the papers involved novel knowledge with which students had some but limited prior experience. Detailed information about these papers, including the journal, title, framework, length, and JCR classification, can be found in Table S3 (Supporting Information). To ensure the comparability in difficulty, an “Expert Evaluation Questionnaire” was developed, comprising five items rated on a 5‐point Likert scale to evaluate content complexity and overall difficulty. Four experts in educational neuroscience assessed the papers, and the results of a Friedman's test on the difficulty ratings indicated no significant differences in difficulty between the materials (*χ^2^* = 3.194, *p* = 0.363). Additionally, the inter‐rater reliability was calculated using intraclass correlation coefficients, which yielded a value of 0.856, indicating a high level of agreement among experts. The four papers were randomly assigned to the four lessons of two conditions.

A within‐subject design was employed. Specifically, the same students participated in both the LH and H‐only conditions. In the LH condition, a low‐structured teaching phase was performed first and followed by a high‐structured teaching phase. In the low‐structured teaching phase, students were provided with one of the four papers as the learning materials a week in advance, and requested to recall the contents of the learning materials and report aloud to other members of the learning group immediately before lecturing. This phase lasted about 10 min and was defined as the recalling phase. Students were not allowed to take notes or communicate with each other freely during this phase. Learning tests were conducted before and after this phase (i.e., pre‐test and recalling‐test) to reflect the learning outcomes. Next, the teacher taught the same knowledge to these students in a lecturing mode for 20 min, which was defined as the lecturing phase (Figure 1B). During this phase, the students were requested to listen carefully and no questioning or answering was allowed. They were permitted to take notes but prohibited from interacting with other students. Students completed the lecturing‐test immediately after teacher's lecturing (Figure 1D). In the H‐only condition, the teachers conducted the lecturing in the same way as in the LH condition immediately after the students arrived in the class, which was defined as the lecturing in the H‐only condition (Figure 1D). No learning materials was given to students, nor were there a recalling procedure prior to teacher's lecturing. The pre‐test and lecturing‐test were conducted before and after teacher's lecturing to reflect the student's learning outcome due to lecturing. Finally, the students were additionally requested to freely recall the knowledge immediately after the lecturing phase, generating an HL condition. The requirement of recalling was the same as that in the LH condition. The recalling‐test was conducted immediately after the recalling phase.

### Behavioral Tests on Learning Outcomes

There were 18 multiple‐choice items in each of the test on learning outcome. According to Bloom's taxonomy,^[^ ^49^ ^]^ the test covers three levels of learning outcomes such as remembering, understanding, and applying, with each level involving six questions (see sample questions in Table S4, Supporting Information). To assess the construct validity of the questionnaire, an exploratory factor analysis was conducted. The results showed that the Kaiser–Meyer–Olkin measure of the sampling adequacy reached 0.648 (ranging from 0.648 to 0.685 across conditions) and Bartlett's tests of sphericity were significant for both conditions (*ps* < 0.001). These metrics suggest that the data were suitable for factor analysis despite the small sample size. Further, three factors were extracted based on eigenvalues greater than 1 and the theoretical assumptions of Bloom's taxonomy. The eigenvalue ranges for these three factors were 3.247–3.673, 1.107–1.614, and 0.935–0.982, respectively. Although the third factor's eigenvalue was slightly below the traditional standard, it was retained as it added ≈9% of explained variance and was theoretically justified. Together, these factors accounted for 34.919–36.227% of the total variance. While the total variance explained was modest (<50%), it was consistent with theoretical expectations and provided a reasonable basis for further investigation given the exploratory nature of this study. The students were asked to complete each test as quickly as possible for about 10 min. The test scores were converted into percentiles.

### fNIRS Data Acquisition

A noninvasive wearable fNIRS system (BRITE 24, Artinis Medical Systems) was used to measure brain activity from each participant. The same brain regions were measured for both teachers and students. Specifically, two customized probe sets were made, with each set having five emitters and four detectors (3 cm distance, 11 measurement channels). One set covered the frontal cortex, while the other covered the right temporoparietal junction, middle temporal gyrus, and superior temporal gyrus along the Sylvian fissure (Figure 1C). Channel 9 was placed on Fpz and channel 19 on CP6 according to the international 10–20 system.^[^ ^84^ ^]^ The positions of the probe sets were checked and adjusted before the experiment to ensure consistency across all participants.

To confirm the anatomical locations of the optode probes, magnetic resonance imaging (MRI) data were obtained from two participants (one male and one female) who wore plastic caps on which the probes’ true positions had been marked using vitamin E balls, with a high‐resolution, T1‐weighted, magnetization‐prepared, and rapid gradient echo sequence (time repetition = 2,530 ms; time echo = 3.30 ms; flip angle = 7°; slice thickness = 1.3 mm; in‐plane resolution = 1.3 × 1.0 m^2^; and number of interleaved sagittal slices = 128). Statistical parametric mapping 12 (Wellcome Department of Cognitive Neurology, London, UK) was used to normalize the MRI data to the standard Montreal Neurological Institute (MNI) coordinate space.^[^ ^85^ ^]^ The MNI coordinates of channels were generated according to the automated anatomical labelling template and Brodmann area using the NIRS_SPM toolbox (see Table S1, Supporting Information).^[^ ^86^, ^87^ ^]^ Based on this information, the authors were able to check the consistency between the probes’ true positions and the expected anatomical positions and to adjust the probes’ true positions. This procedure was repeated several times until the true positions and the expected positions reached a high level of consistency (i.e., the localization probability reported by NIRS_SPM >50%). In addition, these participants did not participate in the current fNIRS experiment.

The optical density of near‐infrared light (760 and 850 nm) was measured at a sampling rate of 10 Hz. Based on the modified Beer–Lambert Law, changes of the oxy‐hemoglobin (HbO) and deoxy‐hemoglobin concentrations were obtained by measuring the absorption changes of fNIRS light after its transmission through the tissue. This study focused only on the changes in the HbO concentration, which was demonstrated to be the most sensitive indicator of changes in the regional cerebral blood flow in fNIRS measurements.^[^ ^88^ ^]^

### Behavioral Data Analyses on Learning Outcomes

The percentile of correct answers was calculated and used as the test score. To test whether the LH condition led to a better learning outcome than did the H‐only condition, a LME model was applied to the test score, with condition as a fixed variable and the identities of lesson, group, and student as random variables, while age and SES as covariates. Here age and SES were included as they have been considered the two most important factors contributing to academic achievement but were irrelevant to this study.^[^ ^89^, ^90^ ^]^ The difference in learning outcomes from pre‐test to lecturing‐test between the LH condition and the H‐only condition was used as the dependent variable, which allowed us to compare the learning outcomes between these two conditions. Additionally, the learning outcome of lecturing‐test (corresponding to the LH condition) or recalling‐test (corresponding to the HL condition) minus pre‐rest, was also compared using the LME method.

### fNIRS Preprocessing

The fNIRS data were checked for data quality using a running‐window procedure.^[^ ^91^, ^92^ ^]^ Specifically, the mean and standard deviation (SD) were calculated within a time window of 10 s. Data points falling beyond the mean ± 3 SD were defined as artifacts. A measurement channel was labeled as bad and was excluded if the percentage of time points with suspected motion artifacts exceeded 5% for the entire time course. A participant was excluded if >30% of the channels were labeled as bad. Based on these criteria, the mean percentage of artifacts across all channels and participants was 1.30%, and no participants were removed during this procedure.

Next, the first and last 15 s of data in each condition were removed to obtain data within the steady state period. Homer3 functions were used to preprocess the data.^[^ ^93^ ^]^ Specifically, motion artifacts were detected and corrected using the discrete wavelet transformation filter procedure,^[^ ^94^ ^]^ and global physiological noises, like skin blood flow, were removed by using principal component analysis (PCA) with an 80% variance threshold.^[^ ^95^ ^]^ Although there have been suggestions on the use of short‐distance channel to remove global physiological noises,^[^ ^96^ ^]^ previous studies have shown that the PCA approach has a comparable performance with the short‐channel approach,^[^ ^97^ ^]^ and the additional use of short‐distance channel will raise difficulty was not suitable for data collection from many students in the real classroom. A band‐pass filter was used to remove high‐ and low‐frequency noises (0.01–0.5 Hz). This band has been most frequently used in previous fNIRS studies.^[^ ^46^ ^]^ Finally, data were *z*‐scored for each participant.

### RSA—Knowledge Segmentation

The audio recordings of each teacher's lecture in the lecturing phase and each student's recalling in the recalling phase were transcribed into text using the iFLYTEK tool (https://www.iflyrec.com/; Figure 2A). Each transcript was manually checked and preprocessed in the following steps. 1) Removal of stop words. Stop words, which were assumed to be semantically uninformative (e.g., “ah,” “huh,” “umm,” “oh,” “seems,” “well”), were filtered out from the text.^[^ ^98^ ^]^ 2) Removal of irrelevant information. Irrelevant utterances, which often appeared at the beginning or end of a sentence and played a role in an attempt to search memory or in marking the end of a production (e.g., “I'll start first,” “I'm done”), were removed.^[^ ^18^ ^]^ Additionally, for students’ recalling, utterances that were factually incorrect (e.g., confabulation) or irrelevant information were excluded. 3) Uniform terminology. Terms with similar meanings were standardized. For example, “interpersonal neural synchronization” was uniformly described as “brain synchronization” in this study, although it might also be referred to as “inter‐brain neural synchronization”; “inter‐brain coupling.”

Next, these transcripts were segmented into minimal knowledge events and entered into the following analyses. To this end, four additional participants—Ph.D. candidates majoring in cognitive neuroscience—were recruited as coders. These coders were unaware of the experimental purpose. They were asked to identify and mark the boundaries of knowledge events independently, following the instructions outlined in the Supporting Information.^[^ ^18^, ^99^ ^]^ The inter‐rater reliability was quantified using ICC, which reached a high level of 0.844. Finally, the four coders convened to identify and resolve any discrepancies through discussion until a consensus was arrived. The segmentation results, based on this consensus, were used for the subsequent analysis. In the end, the duration of knowledge events ranged from 6 to 200 s (mean = 52.741 s) in teachers’ lecturing and from 6 to 88 s (mean = 18.663 s) in students’ recalling.

### Knowledge Matrix

The matrix for knowledge event of teacher's lecturing was next built. Specifically, LDA was used to obtain the vectorized semantic features of each knowledge event.^[^ ^51^ ^]^ Here the semantic features of each knowledge event were transformed into a 4267‐dimensional vector based on topic model of web pages in LDA (https://github.com/baidu/Familia; Figure 2A, left panel). The model was trained on tens of millions of webpage data, with a vocabulary size of more than 280 000, and the number of model topics set to 4267. By calculating the cosine similarity between the LDA vectors of each pair of knowledge events, an event‐by‐event knowledge matrix was obtained (Figure 2A).

It should be noted that the matrix reflected the structure of knowledge. To visualize the knowledge structure, the vectors of the knowledge event were transformed into a graph (Figure 2A), in which knowledge events (nodes) formed connections with each other (edges), and the connection strength between a pair of knowledge events (edge weight) was determined by their semantic similarity. The node degree of each event was the mean of the similarity value of all edges connected to the node in the graph. For students’ recalling, the same procedures were applied, except that the knowledge event matrix was generated for each student and then averaged across all students, to obtain a group‐level matrix.

### Brain Activity Matrix

To perform RSA, the event‐by‐event brain activity matrix for each student in each teaching phase was generated. To this end, an inter‐subject Pearson correlation method was employed to avoid aliasing effect of autocorrelation in the hemoglobin signals of the same participant. Specifically, the preprocessed fNIRS signal corresponding to each knowledge event was extracted. For participant *i* and *j*, an event *m* in participant *i* was correlated to event *n* in participant *j* and vice versa. The correlation was averaged between *r*_(participant_ *_i_*_event_ *_m_*_, participant_ *_j_*_event_ *_n_*_)_ and *r*_(participant_ *_j_*_event_ *_m_*_, participant_ *_i_*_event_ *_n_*_)_ to index the similarity between the two events. This procedure was repeated by correlating event *m* of participant *i* with all other events of participant *j*, resulting in event‐by‐event brain activity similarity matrices for participant *i* and *j*. This procedure was further repeated between participant *i* with all other participants (i.e., *N –* 1), generating *N –* 1 brain activity matrices for participant *i* with a size of *M* × *M*, where *N* is the total number of students and *M* is the number of knowledge events. These matrices were then averaged across *N –* 1 to obtain a single matrix for each channel of participant *i* in each teaching phase (Figure 2B). The above procedure was applied to each participant (*N*).

### Correlation between Knowledge and Brain Activity Matrixes

The representational similarity was measured by computing the Spearman correlation between the lower triangle of the knowledge matrix and the lower triangle of the brain activity matrix within each student. It was worth noting that the brain matrix of each student was asymmetric to the group‐average knowledge matrix during the recalling phase, so the intersection of the element positions of the two lower triangular matrices could be found and the corresponding elements in both matrices could be extracted before doing correlation. The correlation coefficients were averaged across students to obtain a single value for each channel within each teaching phase.

A permutation test was performed by randomly shuffling the phases of fNIRS signals by 1000 times for each participant within each teaching phase. For each iteration, the brain matrix was calculated and correlated to the knowledge matrix. This procedure generated a null distribution of the representation similarity, and a one‐tailed *p*‐value was obtained as the proportion of values from the null distribution equal to or greater than the actual representational similarity (one tailed significance test: *p* = (1 + number of null *r* values ≥ empirical *r*)/(1 + number of permutations)). The issue of multiple comparisons across channels was corrected using FDR method.

### Boundary Effect Analysis

A 15‐s window was selected for the boundary and non‐boundary effect calculation to capture most of the boundary‐related signals based on previous study.^[^ ^58^, ^60^ ^]^ The brain signals have been shifted forward by 6 s to account for the hemodynamic response delay. The boundary periods were defined as the first 15 s following the offset of each knowledge event. The non‐boundary periods were defined as the middle 15 s of each knowledge event. The boundary pattern was defined as the mean brain activity averaged across boundary periods, while the non‐boundary pattern was defined as the mean brain activity averaged across non‐boundary periods (Figure 3A). The difference between boundary and non‐boundary patterns was used to index the strength of the boundary effect. However, the duration for both boundary and non‐boundary patterns was set to 6 s during the recalling phase because of the shorter duration of the knowledge events recalled by students. Finally, the difference between boundary and non‐boundary pattern was compared using paired‐sample *t*‐test on the significant channel that survived the RSA.

### Brain Decoding Analysis

To decode the knowledge structure from brain activity, a leave‐one‐dyad–out regression modeling approach was employed.^[^ ^100^ ^]^ Specifically, 1) According to the RSA procedure (Figure 2B), the brain matrix for each possible pair of students were obtained using the inter‐subject Pearson correlation method (see above). The brain matrices from all potential pairs of students were taken as a population *G*. 2) *G* was split into a training set (*G* – 1 pairs) and a testing set (1 pair). The brain matrices in the training set were averaged, and used to train the regression model. In this model, brain activity matrix was the independent variable, while the knowledge matrix taught by the teacher was the dependent variable. 3) After obtaining the optimal parameters, the testing set (i.e., the held‐out pair of students) was used to test the regression model and generate a predicted knowledge matrix. 4) Steps 2–3 were repeated *G* times. Thus, for each student, *N* – 1 predicted knowledge matrices were obtained and averaged, generating a single predicted knowledge matrix for each student (Figure 4A). 5) Spearman correlation was calculated between the predicted and original knowledge structure. The correlation coefficients were further converted into Fisher *z* to have a normal distribution. 6) A one‐sample *t*‐test was conducted on the *z*‐values across students against zero.

To test individual variance in knowledge construction, pISC was computed based on the predicted knowledge matrix. The Pearson correlation between the predicted knowledge matrix of each student and that of all other students was computed, generating *N* – 1 correlation coefficients. The coefficients were further averaged across *N –* 1 iterations, obtaining an pISC value for each student. The pISC value was converted into Fisher *z* and tested between the LH and H‐only conditions using a paired‐sample *t*‐test.

### Correlation between Brain Representation and Learning Outcome

To investigate whether the MFC's activity correlated with each level of students’ learning outcomes (i.e., remembering, understanding, and applying) under the two conditions, a partial Pearson correlation approach (one‐tailed, *p* < 0.05), controlling for students’ age and SES, was employed. Specifically, for the LH condition, the MFC's representation of knowledge during the lecturing phase with the learning outcomes at each level (i.e., overall outcomes: lecturing‐test minus pre‐test; recalling phase's outcomes: recalling‐test minus pre‐test; lecturing phase's outcomes: lecturing‐test minus recalling‐test) was correlated. For the HL condition, the TPJ's knowledge representation during the lecturing phase and the MFC's knowledge representation during the recalling phase with the learning outcomes at each level (i.e., overall outcomes: recalling‐test minus pre‐test; lecturing phase's outcomes: lecturing‐test minus pre‐test; recalling phase's outcomes: recalling‐test minus lecturing‐test) was correlated.

To test the correlation between recalling performance and neural representation of knowledge in details, the recalling performance for each student was qualified using binary encoding and pISC method in the LH condition. Specifically, binary encoding (0,1) procedure was employed, where correctly recalled knowledge events were coded as 1 and other events as 0, obtaining a performance vector for each student. Next, the similarity between each student and all of other students on the recalling performance vector was computed using the Jaccard similarity method and averaged the similarity across all potential correlations, deriving a behavioral pISC value for each student, which reflected the consistency (approaching 1) or inconsistency (approaching 0) of the recalling performance among students. Similarly, the Pearson correlation can be used to calculate the similarity between each student and other students in the neural representation of knowledge structure during the lecturing phase, and generated a neural pISC value for each student. Finally, the correlation between the recalling performance and the neural representation of knowledge structure was calculated using Pearson correlation.

### Teacher–Student Neural Synchronization

According to the previous RSA analysis, the neural representation of knowledge structure on each channel for each student was obtained. To examine whether the teacher predicted the knowledge status of students in advance during the teaching process, the time courses of the teacher's brain activity were shifted forward or backward with a step = 2 s, relative to that of students’ brain activity. At each time‐lag, the neural representation of knowledge structure for the teacher was calculated, resulting in corresponding brain matrix for each channel. To compute the teacher–student neural synchronization in representing the knowledge structure, the Spearman correlation between the teacher's and students’ brain matrices for all possible teacher–student CH combinations (i.e., 22 × 22 = 484) was calculated. The minimum size of the time‐lag was determined according to the authors’ previous study,^[^ ^42^ ^]^ while the maximum size of the time‐lag was determined when the correlation coefficients started to decline. Thus, a range of 6–14 s was reported.

A permutation test was conducted by randomly shuffling the phases of fNIRS signals by 1000 times for each student. After each shuffle, the brain matrix across students were recalculated and they were correlated with the teacher's brain matrix at each time lag. This process was repeated 1000 times to generate a null distribution to obtain a one‐tailed significant *p*‐value, and the results for all channel combinations were corrected for multiple comparisons using the FDR method across all time‐lags at *p* < 0.05 level.

## Conflict of Interest

The authors declare no conflict of interest.

## Author Contributions

C.L. and X.F. conceived the experiment. X.F., Z.M., and M.P. performed the research. X.F., X.X., and J.J. analyzed the data. X.F. drafted the paper. C.L., X.F., and Y.Z. revised the paper.

## Supporting information



Supporting Information

## Data Availability

All data needed to evaluate the conclusions in the paper are present in the main text or the Supporting Information. The raw data are available from the corresponding author upon request. All analyses of fNIRS data were performed using MATLAB R2021a with standard functions and toolboxes. Machine learning model analyses were carried out using Python 3.7.9, also utilizing standard functions and toolboxes. All codes used are available upon request.
