# Supplementary material for: A Rapid Cortical Learning Process Supporting Students’ Knowledge Construction During Real Classroom Teaching
Source: Adv Sci (Weinh). 2025 Feb 7;12(18):2416610. doi: 10.1002/advs.202416610 (PMC12079370; doi:10.1002/advs.202416610)
Supplement: Supplementary file 1 — Supporting Information [file ADVS-12-2416610-s001.docx]

Supporting information for

**A Rapid Cortical Learning Process Supporting Students’ Knowledge Construction during Real Classroom Teaching**

Xiaodan Feng, Xinran Xu, Zhaonan Meng, Jiahao Jiang, Miao Pei, Yonghe Zheng, Chunming Lu^*^

**Instructions for Marking Knowledge Events.**

Please carefully listen to the audio and segment the content into knowledge events based on the audio’s structure. Assign a name to each knowledge event. If you believe a sentence introduces or explains a different concept, theory, method, etc., compared to the previous sentence, mark the end of the sentence with an Arabic numeral (e.g., 1, 2, 3) and assign a name to the knowledge event. The smallest unit for marking is a sentence, meaning you can only mark at the end of a sentence. Explanations and examples of the same concept, theory, or method should be grouped under the same knowledge event. If you are uncertain whether two events belong to the same knowledge event, or if there is overlap or hierarchical relationships between knowledge events, segment them into sub-concepts as much as possible without affecting comprehension. For example, if the study includes three hypotheses (Hypothesis 1, Hypothesis 2, Hypothesis 3), segment them into three knowledge events. Summarizing statements that synthesize multiple previous knowledge events can also be considered a new knowledge event, as they represent a different hierarchical relationship from the previous events.

**Expert Evaluation Questionnaire**

Instructions: Please rate your level of agreement with each statement on a 5-point Likert scale: 1 = strongly disagree, 2 = disagree, 3 = neutral, 4 = agree, 5 = strongly agree.

Section 1: Content Complexity

1. The paper introduces theoretical frameworks and new concepts that require careful thinking to understand.
2. The data analysis and interpretation in the paper involve complex statistical or conceptual methods.
3. The methodology presented in the paper poses a significant challenge to learners when studying and applying to personal data.
4. The paper requires a significant background in educational neuroscience to fully understand its content.

Section 2: Overall Difficulty

1. Overall, I found this paper difficult to read and understand.


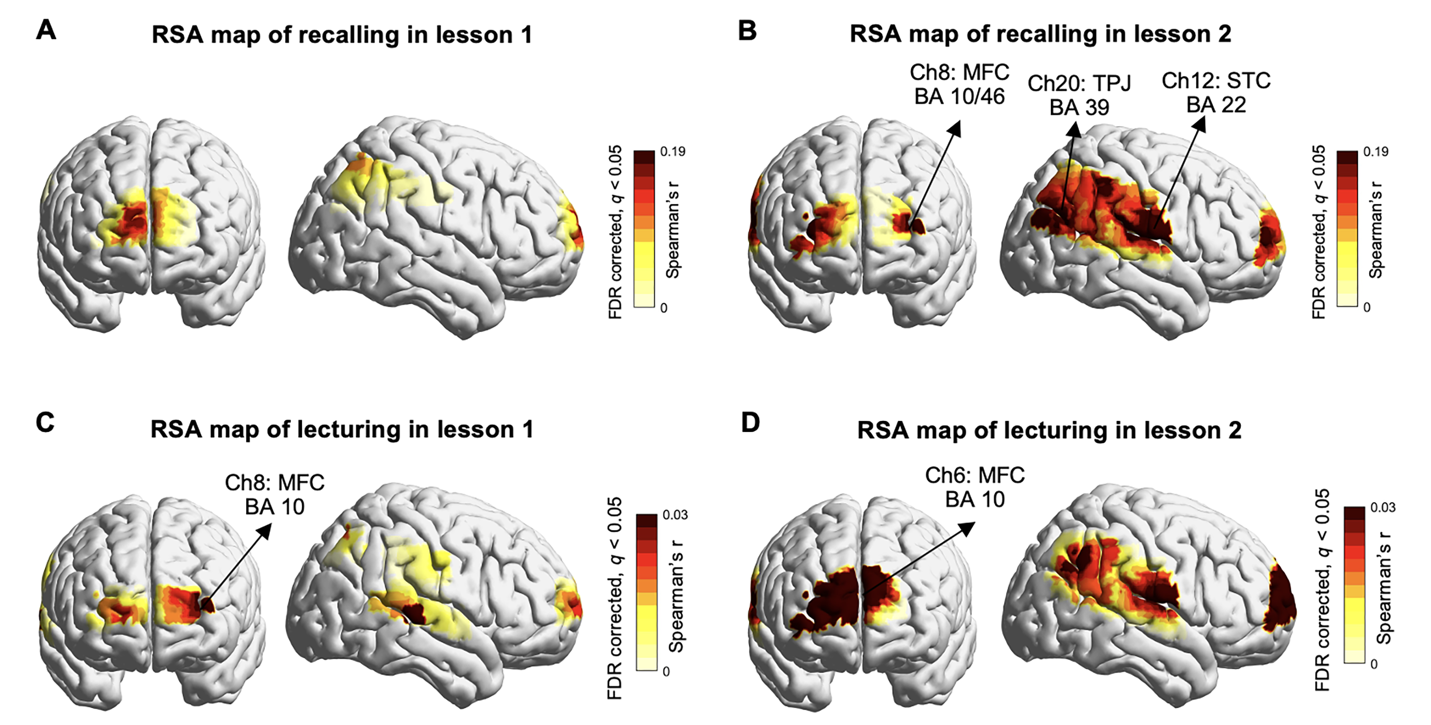


Figure S1. RSA results in the LH condition. A) In lesson 1, the RSA map showed the largest *r* values in the MFC (CH 5) across all CHs during the recalling phase, but it did not survive the FDR correction at *p* < 0.05 level. B) In lesson 2, the RSA map showed that knowledge was significantly represented in the MFC (CH 8), TPJ (CH 20) and STC (CH 12) during the recalling phase. C) In lesson 1, the significant representation of knowledge was found in the MFC covered by CH8 during the lecturing phase. D) In lesson 2, the significant representation of knowledge was found in the MFC covered by CH6 during the lecturing phase.


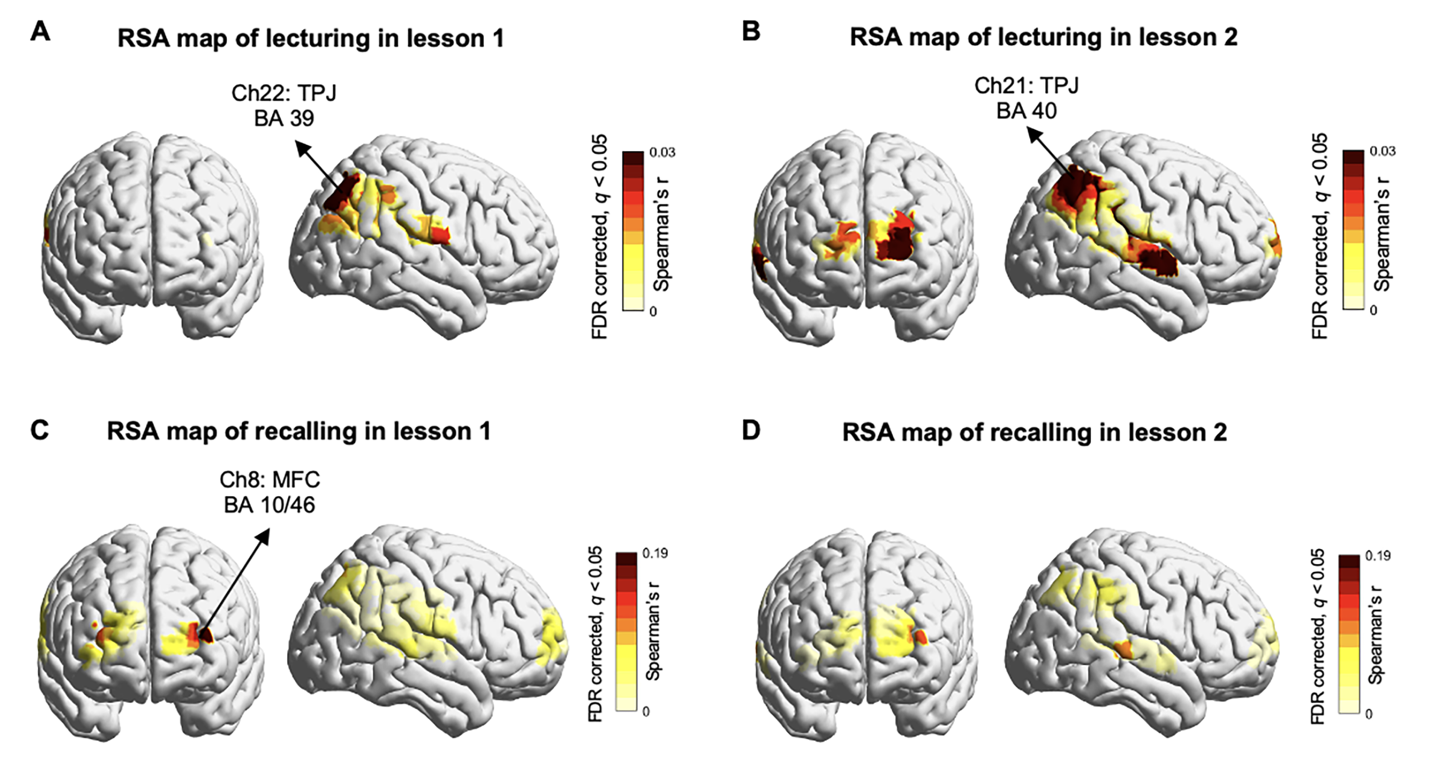


Figure S2. RSA results in the HL condition (including H-only condition). A) In lesson 1, the RSA map showed that knowledge was significantly represented in the TPJ (CH 22) during the lecturing phase. B) In lesson 2, the significant representation of knowledge was found in the TPJ (CH 21) during the lecturing phase. C) In lesson 1, the significant representation of knowledge was found in the MFC covered by CH 8 during the recalling phase. D) In lesson 2, the RSA map showed the largest *r* values in the MFC (CH 8) across all CHs during the recalling phase, but it did not survive the FDR correction at *p* < 0.05 level.


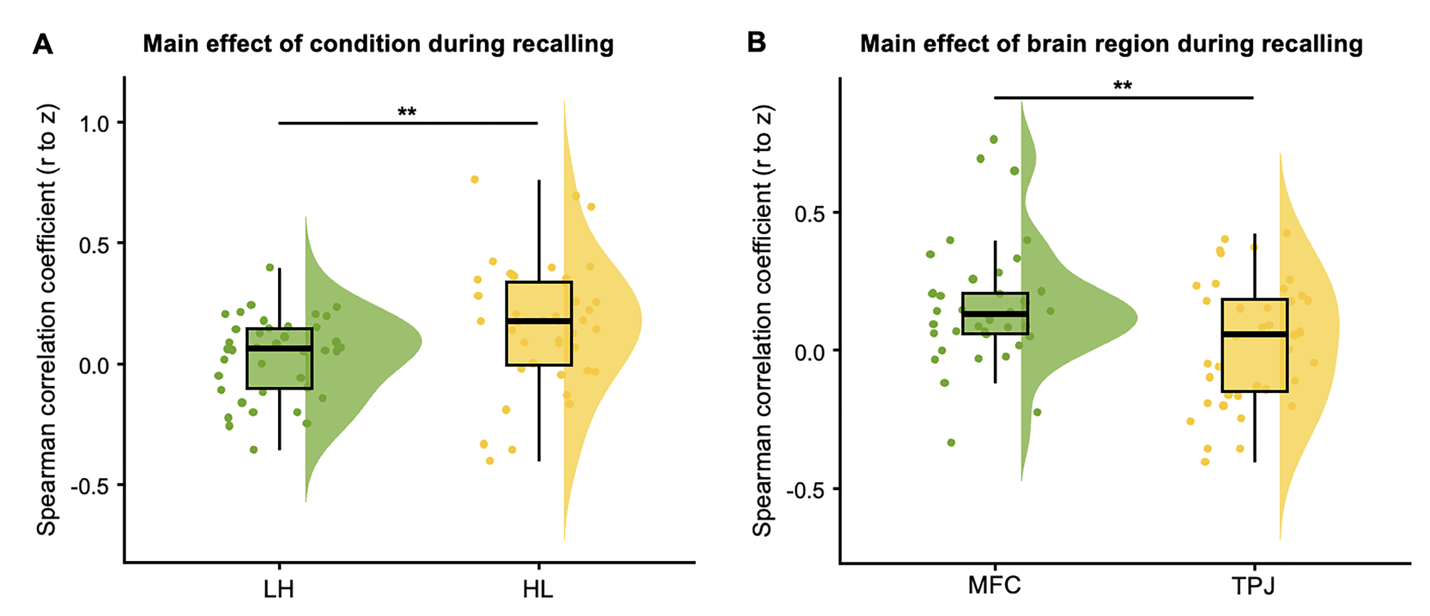


Figure S3. Results of the condition × brain region ANCOVA during the lecturing phase using the LME method. There were significant main effects of both condition (A) and brain region (B).


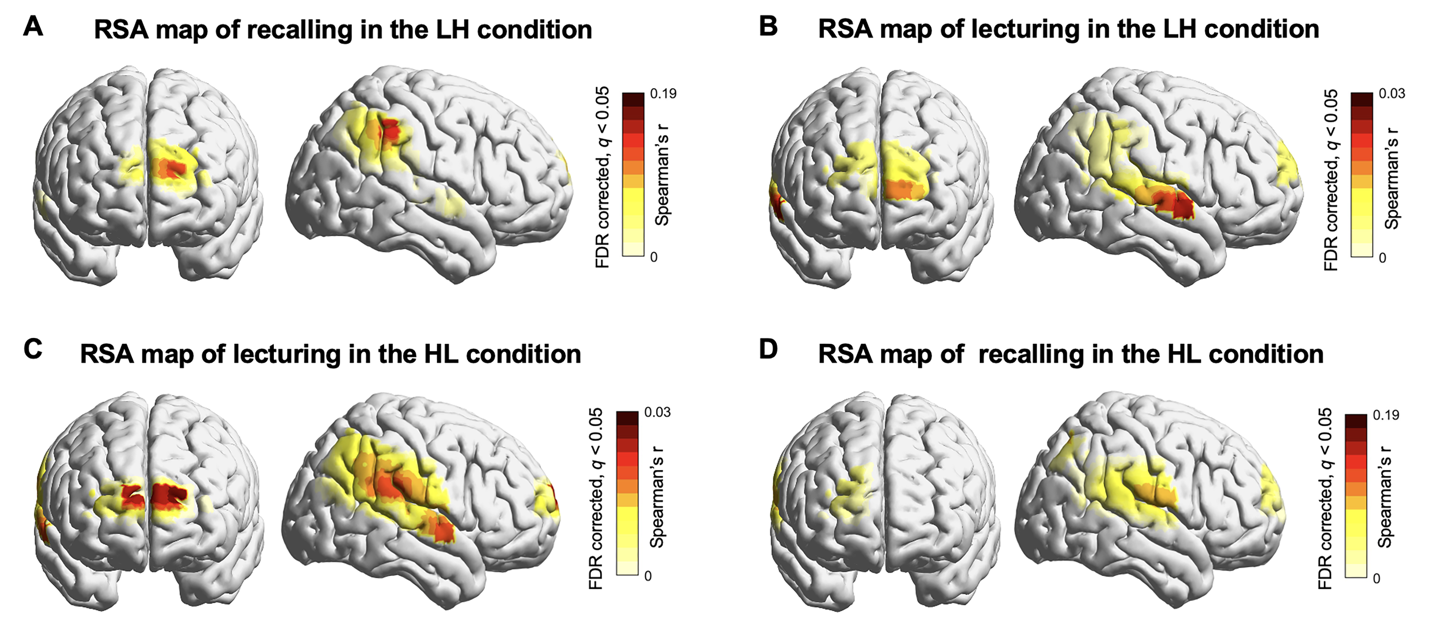


Figure S4. The results of RSA validation through random permutation. (A-D) The random data did not have any significant results either during the lecturing phase or recalling phase of both conditions.

Table S1 MNI coordinates of each measurement channel.

|  | MNI coordinates | | | Region name and percentage of overlap | |
| --- | --- | --- | --- | --- | --- |
| CH | x | y | z | Anatomical Labeling | Brodmann Area |
| 1 | 34 | 61 | 21 | Frontal Superior Cortex (0.328)  Frontal Middle Cortex (0.672) | BA 10 (0.425)  BA 46 (0.575) |
| 2 | 45 | 58 | 7 | Frontal Middle Cortex (0.863) | BA 10 (0.398)  BA 46 (0.602) |
| 3 | 25 | 71 | 11 | Frontal Middle Cortex (0.986) | BA 10 (0.942) |
| 4 | 21 | 61 | 34 | Frontal Superior Cortex (0.684)  Frontal Middle Cortex (0.229) | BA 9 (0.474)  BA 10 (0.383) |
| 5 | 13 | 70 | 22 | Frontal Superior cortex (0.428)  Frontal Superior Medial cortex (0.569) | BA 10 (1.000) |
| 6 | 0 | 61 | 33 | Frontal Superior Medial cortex (0.735) | BA 9 (0.353)  BA 10 (0.647) |
| 7 | -22 | 62 | 29 | Frontal Superior Cortex (0.845) | BA 10 (0.456)  BA (0.353) |
| 8 | -34 | 62 | 17 | Frontal Middle Cortex (0.677)  Frontal Superior Cortex (0.323) | BA 10 (0.542)  BA 46 (0.458) |
| 9 | -2 | 69 | 10 | Frontal Superior Medial cortex (0.965) | BA 10 (1.000) |
| 10 | -13 | 69 | 23 | Frontal Superior Cortex (0.717) | BA 10 (1.000) |
| 11 | -23 | 70 | 10 | Frontal Superior Cortex (0.973) | BA 10 (0.928) |
| 12 | 70 | -11 | 16 | Postcentral Cortex (0.433)  Temporal Superior Cortex (0.387) | BA 22 (0.570)  BA 43 (0.393) |
| 13 | 70 | -8 | -7 | Temporal Superior Cortex (0.567)  Temporal Middle Cortex (0.433) | BA 21 (0.886) |
| 14 | 72 | -32 | 12 | Temporal Superior Cortex (0.838) | BA 22 (0.913) |
| 15 | 68 | -15 | 35 | Postcentral Cortex (0.506)  Supramarginal Gyrus (0.494) | BA 43 (0.380)  BA 2 (0.325) |
| 16 | 70 | -33 | 33 | Supramarginal Gyrus (0.969) | BA 2 (0.349)  BA 40 (0.538) |
| 17 | 65 | -36 | 48 | Supramarginal Gyrus (0.696)  Parietal Inferior Cortex (0.304) | BA 40 (0.875) |
| 18 | 65 | -54 | 29 | Angular Gyrus (0.580)  Temporal Superior Cortex (0.247) | BA 22 (0.539)  BA 39 (0.413) |
| 19 | 59 | -56 | 46 | Parietal Inferior Cortex (0.731)  Angular Gyrus (0.235) | BA 40 (0.549)  BA 39 (0.451) |
| 20 | 51 | -72 | 41 | Angular Gyrus (0.966) | BA 39 (0.936) |
| 21 | 48 | -55 | 59 | Supramarginal Gyrus (0.763) | BA 40 (0.783) |
| 22 | 41 | -70 | 54 | Angular gyrus (0.959) | BA 39 (0.717) |

Note: The numbers in the bracket indicate the location likelihood of brain areas.

Table S2 Descriptive statistics for the two lessons in the study.

|  | **Lesson 1** | | **Lesson 2** | |  |  |  |  |  |
| --- | --- | --- | --- | --- | --- | --- | --- | --- | --- |
| **Variable** | **Mean** | **SD** | **Mean** | **SD** | ***t*** | **df** | ***p*** | **Cohen’s *d*** | **95% CI (low, high)** |
| **Age** | 25.462 | 3.307 | 25.500 | 2.390 | 0.029 | 19 | 0.978 | 0.013 | [-0.893, 0.868] |
| **SES** | 4.846 | 1.676 | 5.375 | 1.768 | 0.688 | 19 | 0.500 | 0.309 | [-1.192, 0.586] |
| **Baseline-test** | 36.769 | 16.249 | 40.250 | 13.957 | 0.502 | 19 | 0.622 | 0.225 | [-1.106, 0.664] |

Table S3 Description of learning materials (four scientific papers).

| **Condition** | **Lesson** | **Journal** | **Title** | **Framework** | **Length** | **JCR** |
| --- | --- | --- | --- | --- | --- | --- |
| LH | 1 | Child development | Interpersonal neural synchrony during father–child problem solving: an fNIRS hyperscanning study. | 1) Introduction  2) Methods  3) Results  4) Discussion | 16 pages | Q1 |
|  | 2 | Cortex | The effects of interaction quality on neural synchrony during mother-child problem solving. |  | 15 pages | Q1 |
| H-only | 1 | Human brain mapping | Enhancement of teaching outcome through neural prediction of the students’ knowledge state. |  | 12 pages | Q1 |
|  | 2 | Neuroimage | Instructor-learner brain coupling discriminates between instructional approaches and predicts learning. |  | 13 pages | Q1 |

Table S4 Example items of the test at the Remember, Understanding and Applying level, respectively.

| **Tests** | **Examples** |
| --- | --- |
| Remember | Who is the first author of this study? |
| Understanding | To test the prediction-transmission hypothesis, this study conducted a time-lag analysis on brain synchronization between teachers and students. Which of the following descriptions on the results is correct? |
| Applying | Which of the following options is not in line with the purpose of this study? |
